# Genome-wide discovery and validation of diagnostic DNA methylation-based biomarkers for hepatocellular cancer detection in circulating cell free DNA

**DOI:** 10.7150/thno.35573

**Published:** 2019-09-25

**Authors:** Ryan A. Hlady, Xia Zhao, Xiaoyu Pan, Ju Dong Yang, Fowsiyo Ahmed, Samuel O. Antwi, Nasra H. Giama, Tushar Patel, Lewis R. Roberts, Chen Liu, Keith D. Robertson

**Affiliations:** 1 Department of Molecular Pharmacology and Experimental Therapeutics, Mayo Clinic, Rochester, MN, USA; 2 Division of Gastroenterology and Hepatology, Department of Medicine, College of Medicine, Mayo Clinic, Rochester, MN, USA; 3 Department of Health Sciences Research, Mayo Clinic, Jacksonville, FL, USA; 4 Department of Transplantation, Mayo Clinic, Jacksonville, FL, USA; 5 Department of Pathology and Laboratory Medicine, New Jersey Medical School, Rutgers, The State University of New Jersey, Newark, NJ, USA

**Keywords:** liver cirrhosis, hepatocellular cancer, biomarker, epigenetics, cfDNA, DNA methylation

## Abstract

Hepatocellular carcinoma (HCC), the most prevalent form of liver cancer, is growing in incidence but treatment options remain limited, particularly for late stage disease. As liver cirrhosis is the principal risk state for HCC development, markers to detect early HCC within this patient population are urgently needed. Perturbation of epigenetic marks, such as DNA methylation (5mC), is a hallmark of human cancers, including HCC. Identification of regions with consistently altered 5mC levels in circulating cell free DNA (cfDNA) during progression from cirrhosis to HCC could therefore serve as markers for development of minimally-invasive screens of early HCC diagnosis and surveillance.

**Methods**: To discover DNA methylation derived biomarkers of HCC in the background of liver cirrhosis, we profiled genome-wide 5mC landscapes in patient cfDNA using the Infinium HumanMethylation450k BeadChip Array. We further linked these findings to primary tissue data available from TCGA and other public sources. Using biological and statistical frameworks, we selected CpGs that robustly differentiated cirrhosis from HCC in primary tissue and cfDNA followed by validation in an additional independent cohort.

**Results**: We identified CpGs that segregate patients with cirrhosis, from patients with HCC within a cirrhotic liver background, through genome-wide analysis of cfDNA 5mC landscapes. Lasso regression analysis pinpointed a panel of probes in our discovery cohort that were validated in two independent datasets. A panel of five CpGs (cg04645914, cg06215569, cg23663760, cg13781744, and cg07610777) yielded area under the receiver operating characteristic (AUROC) curves of 0.9525, 0.9714, and 0.9528 in cfDNA discovery and tissue validation cohorts 1 and 2, respectively. Validation of a 5-marker panel created from combining hypermethylated and hypomethylated CpGs in an independent cfDNA set by bisulfite pyrosequencing yielded an AUROC of 0.956, compared to the discovery AUROC of 0.996.

**Conclusion**: Our finding that 5mC markers derived from primary tissue did not perform well in cfDNA, compared to those identified directly from cfDNA, reveals potential advantages of starting with cfDNA to discover high performing markers for liquid biopsy development.

## Introduction

Hepatocellular carcinoma (HCC), the most common primary liver tumor, is the 2^nd^ leading cause of cancer-related deaths worldwide, and is the 6^th^ leading cancer killer in the United States. Both incidence and deaths from HCC have been increasing in the United States over the last 20 years, driven largely by undiagnosed hepatitis C viral (HCV) infection and obesity-driven non-alcoholic fatty liver disease (NAFLD). Known etiologic risk exposures directly contribute to development of liver cirrhosis, including chronic alcohol abuse and hepatitis B/C viral infections, and in more recent years the growing epidemic of metabolic syndrome and NAFLD 1. Liver cirrhosis represents the main risk state for HCC development, with 2-4% incidence of HCC per year in patients with liver cirrhosis 2. Thus, there is an urgent unmet need for biomarkers that can detect early HCC development in a cirrhotic liver background.

HCC is a cancer where early detection makes a significant difference; survival rates can be as high as 50% if detected early 3. HCC detection has relied largely on screening high risk groups with serological markers such as alpha-fetoprotein (AFP), followed by imaging of the liver by ultrasound (US) 4, however current professional guidelines have largely abandoned AFP on its own because of its low sensitivity (40-60%) and because many HCCs are AFP-negative 4, 5. US imaging is relatively inexpensive and a less demanding procedure for the patient, however performance of US alone in detecting HCC varies by as much as 23-90% in sensitivity 6. MRI/CT-scan on the other hand, can exceed sensitivity of 80%, but this procedure is typically reserved for only those at highest risk since it is expensive and uncomfortable 6. Even for suspicious lesions detected by MRI/CT, there is ambiguity in the diagnosis ~5-15% of the time 7. It is well known that HCC frequently displays heterogeneous growth patterns and/or cytologic features within a single patient's tumor that is likely driven by heterogeneity at both the genetic and epigenetic levels 8-11. Cirrhotic tissue also presents with significant genetic and epigenetic heterogeneity 12, 13. Heterogeneity within the HCC patient population is likely further compounded by the distinct effects of different etiologic exposures (e.g. HCV versus alcohol). Collectively these aspects of liver cirrhosis and HCC no doubt impede biomarker development, particularly when markers are discovered using a single region of tissue.

Deregulated DNA methylation (5mC) patterns, characterized by global hypomethylation and gene-specific hypermethylation, are a hallmark of tumor cells 14-18. The frequency and number of aberrantly methylated genes increases during liver disease progression and correlates with poor prognosis 19, 20. Hypomethylation also becomes more pronounced with disease progression 16, 21. While particular 5mC signatures have been linked to HCC grade, differentiation status, progression, and survival 16, 22, none have made their way into clinical use. Many additional layers of epigenetic control are deregulated in and contribute to HCC development, particularly those marks regulating enhancer activity 23. While these studies have contributed to our understanding of the epigenetic underpinnings of cirrhosis and HCC and potential disease driver events, they have not yielded markers for early HCC detection.

Circulating cell-free DNA (cfDNA) in plasma is rapidly emerging as a potent non-invasive blood-based biomarker, or liquid biopsy, for early cancer detection, and for monitoring tumor progression, prognosis, and response to treatment 24. In healthy individuals, plasma cfDNA is thought to originate from the hematopoietic cell compartment, but in cancer patients also derives from apoptotic or necrotic processes characteristic of tumor cells with high turnover rates 25, 26. Markers include specific mutations found in tumor cells (*e.g*. EGFR 27) and changes to 5mC patterns (*e.g.* SEPT9 28). Indeed, while not plasma based, a stool-based DNA test for early colon cancer detection currently on the market makes use of both genetic and epigenetic markers 29. A key advantage of DNA methylation as a cfDNA-based marker lies in the tissue specificity of epigenetic marks in general, which in principle permit not only detection of a cancer-specific signature, but also identification of the source tissue. Recent studies highlight this potential for cfDNA methylation, demonstrating that even with cancer-specific epigenetic changes, sufficient cell-type specificity remains to permit identification of cell-of-origin 25. Multiple examples of 5mC markers derived from candidate gene studies support the potential of cfDNA 5mC as a cancer detection/prognosis marker (*e.g*. lung cancer 30 and metastatic breast cancer 31), however the majority of such studies begin with tumor-normal tissue comparisons to generate potential markers, requiring extensive evaluation of their performance in cfDNA and making them subject to intratumoral heterogeneity effects. Genome-wide methods for biomarker development, including CancerLocator and CancerDetector, also start with profiling of primary tissue, potentially missing key markers specific to cfDNA, and also limiting the ability to account for cirrhosis as a reference 32, 33. In the current study, we performed unbiased genome-wide 5mC profiling in cfDNA and primary liver tissues from three independent cohorts comprised of patients with or without HCC, to identify specific CpGs that differentiate between these two disease states. This approach proved to be powerful, as a panel of five hyper- or hypomethylated CpG markers was sufficient to achieve an area under the receiver operating characteristic (AUROC) curve greater than 0.95 in both the discovery cfDNA dataset and two independent primary tissue-based validation cohorts. Interestingly, however, many of the high scoring 5mC marker panels derived from tissue did not perform nearly as well in cfDNA, demonstrating the potential benefit of developing the 5mC panel directly from cfDNA rather than primary tissue. Our cfDNA 5mC markers therefore represent promising early HCC detection markers that merit validation in a larger patient population.

## Methods

### Human subjects and sample acquisition

Methylation data used in this analysis is derived from four cohorts. Two primary liver tissue datasets from public sources 18, 34, normal primary CD4, CD8, and CD19 cells 35, and cfDNA from a cohort of patients collected at Mayo Clinic, Rochester. Patients in tissue set 1 and all cfDNA data (both the Infinium 450k and the pyrosequencing cohorts) had background cirrhosis. Tissue set 1 is comprised of 82 non-tumor (cirrhotic) and 109 HCC (with background cirrhosis) samples from TCGA LIHC and GSE60753. Tissue set 2 includes patients with cirrhosis and lower stages of fibrosis (Ishak scores 0-5 or unknown) derived exclusively from TCGA LIHC with 45 non-tumor and 306 tumor samples. The Mayo Clinic cfDNA cohort analyzed by the Infinium 450k array is comprised of an equal number of patients in the HCC and no HCC groups (22 in each group, all with cirrhosis, Table S1, derived from the International Hepatobiliary Neoplasia Registry and Repository). At the time of plasma collection, patients in the cirrhosis only group had not received any HCC-related treatments and must have had at least two successive imaging sessions without a diagnosis of HCC. The bisulfite pyrosequencing validation cohort included 15 cirrhosis alone and 15 cirrhosis with HCC cfDNA samples. For all cfDNA samples, a total of 2-4mL of plasma (EDTA preserved) was collected and cfDNA isolated using the QIAamp Circulating Nucleic Acid Kit (Qiagen). Additional clinical and demographic parameters on patient samples are provided in Tables S1-2.

### DNA methylation

CfDNA samples are assayed for DNA methylation using the Infinium HumanMethylation450k BeadChip array (450k; Illumina). Primary tissue 450k data including TCGA HCC (LIHC) data is obtained from the GDC legacy archive and previous work from our laboratory 18. Normal leukocyte 450k data is retrieved from NCBI GEO GSE35069 35. Each dataset is normalized and processed independently utilizing the R package 'minfi' following protocols for subset-quantile within array normalization, as previously described 36, 37. Probes from allosomes, those associated with SNPs, and those that did not reach significance in all cfDNA samples are filtered out, leaving a total of 377,286 CpGs for downstream analysis. The resultant beta (β) values are used to identify changes in DNA methylation (Δβ) between groups. All array data has been deposited in NCBI GEO (GSE129374). Bisulfite pyrosequencing was performed on a PyroMark Q24 (Qiagen) following standard protocols 38. In brief, cfDNA was extracted from 2-3mL plasma from 30 patients with cirrhosis, half of which had concurrent HCC. CfDNA was bisulfite treated using the Zymo EZ DNA Methylation kit and amplified by PCR using a biotinylated HPLC-purified reverse primer and standard forward primer (Table S3). The biotinylated strand was captured with streptavidin sepharose HP beads and processed on the PyroMark Q24.

### Software packages and statistical analysis

Differential methylation between non-tumor and tumor samples is determined by both biological (Δβ > |0.1|) and statistical (P < 0.05) cutoffs. Data are visualized using R packages including 'ggplot2', 'heatmap.3', and 'glmnet'. Shrinkage analysis is performed using 'glmnet' and 'HDCI'. Initial filtering of cfDNA data yielded 443 hypermethylated and 1,770 hypomethylated CpGs, which are linked to primary tissue set 1 to ensure that data is available for the same set of CpGs. The hyper- and hypomethylated CpG cohorts are each used for Lasso linear regression with α = 0.05, λmin and 100 bootstraps (Tables S4-5). Weighted CpGs are selected with the lower bound 95% confidence interval for the coefficient > 0 (or upper bound < 0 for hypomethylation), resulting in 13 CpGs for hypermethylation and 10 CpGs for hypomethylation. Stepwise manual recursive partitioning is used to further hone in on CpGs with a maximum of 5 CpGs in a panel to avoid overfitting, similar to other approaches 39. Area under the receiver operating characteristic curve (AUROC) analysis is performed using the R package 'pROC'. Ingenuity Pathway Analysis (IPA, Qiagen) and Genomic Regions Enrichments of Annotation Tool (GREAT 40) is used for gene ontology and comparative analyses.

## Results

### Characterization of samples and genome-wide DNA methylation analysis

**Figure 1:**
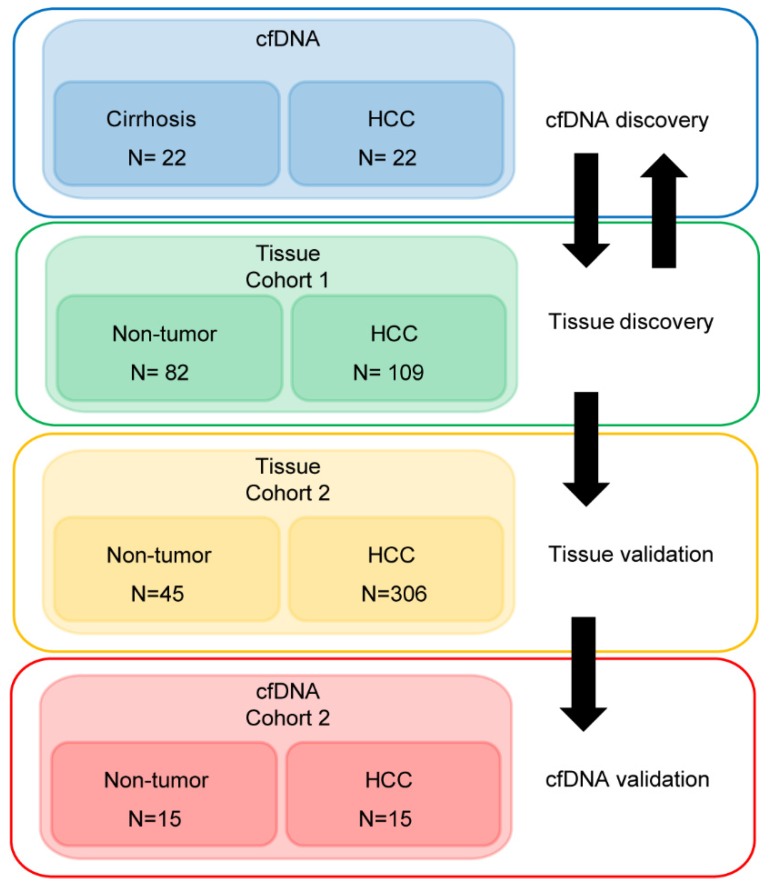
** Approach to identifying DNA methylation-based biomarkers for detection of early HCC in liver cirrhosis patients.** We utilized two strategies to identify DNA methylation-based biomarkers. First, starting with primary tissue to discover markers and then examining their performance in cfDNA, and the converse, starting with cfDNA to discover markers then validating these markers in two independent primary tissue datasets. We then validated a 5-marker panel in 30 independent cfDNA samples by bisulfite pyrosequencing. A total of 586 patient-derived Infinium 450k profiles are assessed for this study.

**Figure 2:**
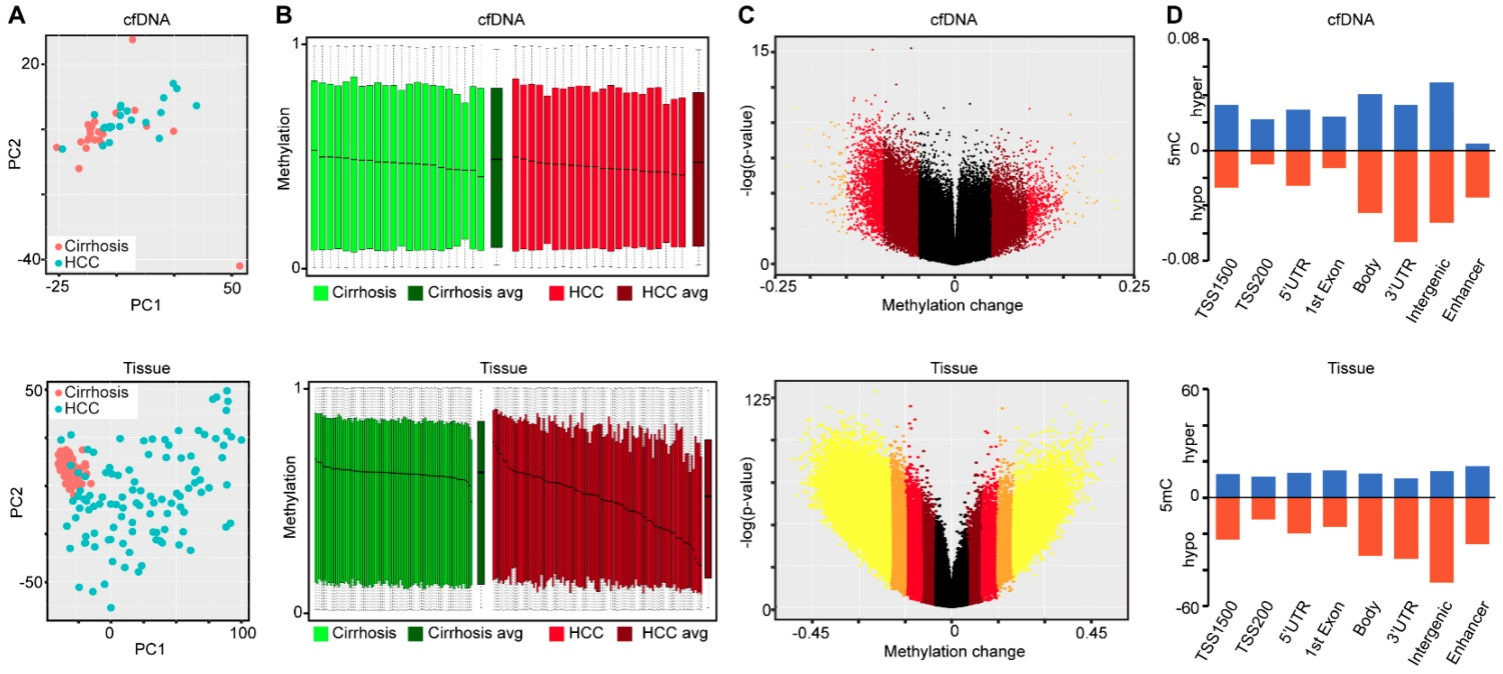
** Characterization of primary tissue- and cfDNA-derived DNA methylation landscapes. A**) Principal component analysis of differentially methylated CpGs in cfDNA (top, n=44; 22 cirrhosis in pink, 22 HCC in blue) and primary tissues (bottom, n=191; 82 cirrhosis in pink, 109 HCC in blue) using all CpGs on the array after QC filtering (including removal of X, Y, and SNP associated probes). **B**) Overall methylation level bar charts (as beta values) for individual cirrhotic (green), average of all cirrhotic (dark green), individual HCC (red), and average of all HCC (dark red) patients derived from cfDNA (top) or primary liver tissues (bottom). **C**) Volcano plots of 5mC changes plotted against -log P values between cirrhotic only and cirrhotic with concurrent HCC patients. Stepwise coloring of changes is based on delta beta values (0.05 increments) with black being below 0.05, dark red between 0.05-0.1, red between 0.1-0.15, orange between 0.15-0.20, and yellow greater than 0.20 in cfDNA (top) and primary tissue (bottom). **D**) Bar charts representing the relative distribution of DNA methylation changes (Δβ > 0.1 tumor vs non-tumor) across the indicated features based on 450k data derived from cfDNA (top) and from primary tissue (bottom). Blue and orange bars represent hypermethylation and hypomethylation events, respectively.

Demographic and clinical information on the cfDNA- and tissue-derived DNA samples used for Infinium 450k array-based DNA methylation analysis are summarized in Figure 1 and Table S1. Figure 1 also shows a flowchart summarizing our analytic approach to marker identification and validation. CfDNA samples were selected based on an even distribution of MELD score between cirrhosis and HCC to adjust for this potential confounding factor that could lead to methylation markers of liver function rather than early HCC detection (Figure S1A, Table S1). CfDNA was prepared from 2-4ml of plasma (yields are summarized in Table S1) and 5mC levels in cfDNA assessed genome-wide using the Illumina '450k' Beadchip array, as described by our laboratory previously 18, 23 and in the methods section. 5mC data from tissue was acquired from TCGA, Reinius *et al*., and previous work by our group, all generated using the 450k array 18, 23, 35 (Figure 1 and Table S1). Principal component analysis using all CpGs on the array that pass quality control (after removal of chromosome X and Y probes) reveals robust segregation between cfDNA derived from patients with cirrhosis and those with HCC (with background cirrhosis, Figure 2A, top), and for cirrhotic and HCC primary tissues (Figure 2A, bottom). The expected tumor global hypomethylation phenotype is observed based on the mean 5mC levels in each group of samples (Figure 2B, darker bars show averages for each sample type/group). Also consistent with prior studies examining 5mC changes in cancer in general, a more modest number of hypermethylation events occur within the 'sea' of hypomethylation changes (Figure 2C). These overall trends are observed in both tissue- and cfDNA-derived 5mC patterns, with the key difference being the much greater total number of events in both directions in cirrhotic versus HCC primary tissue comparisons, relative to cfDNA from the same disease states (compare top and bottom panels and note difference in scale, Figure 2C). The distribution of 5mC changes by genomic feature reveals enrichment of hypermethylation events in promoter regions, while hypomethylation events are more frequent in gene bodies and intergenic regions, consistent with bodies being more heavily methylated in normal cells in general (Figure 2D). These findings demonstrate that cfDNA shows similar overall methylation differences between cirrhotic and HCC states, with the main difference being fewer changes and changes of smaller magnitude, relative to the comparable tissue-based differences.

Since the presence of normal leukocyte-derived DNA may confound interpretation of DNA methylation landscapes derived from cfDNA, we performed PCA on all primary tissue and cfDNA samples, along with purified CD4, CD8, and CD19 5mC landscapes derived from public sources. This analysis reveals that the leukocyte population localizes to a distinct cluster independent from non-tumor and HCC samples (Figure S1B). Further characterization using the 10,000 most variable CpGs across primary tissue and cfDNA demonstrate that cirrhotic and HCC primary tissues cluster independently, as expected, but these disease states cluster less distinctly for 5mC patterns derived from cfDNA (Figure S2). This result is not surprising given that primary tissue samples represent relatively distinct populations of cirrhotic or HCC-derived cells. On the other hand, for patients with HCC, DNA from both cirrhotic liver (which typically comprises a much larger fraction of liver tissue by mass than the tumor) and HCC nodules is presumably shed into the bloodstream, resulting in a mixture of 5mC signals derived from both disease states. This could also explain why fewer CpGs are identified as statistically significantly different in cfDNA relative to primary tissue comparisons (Figure 2C). Despite this, we are able to robustly segregate cirrhosis from HCC states when examining cfDNA samples in the absence of any primary tissue-derived data (Figure 2A, top). This finding suggests that cfDNA, independent of primary tissue, is a viable source of DNA methylation-based markers for the detection of HCC in the setting of liver cirrhosis.

### Identification of DNA methylation markers from primary tissue

**Figure 3:**
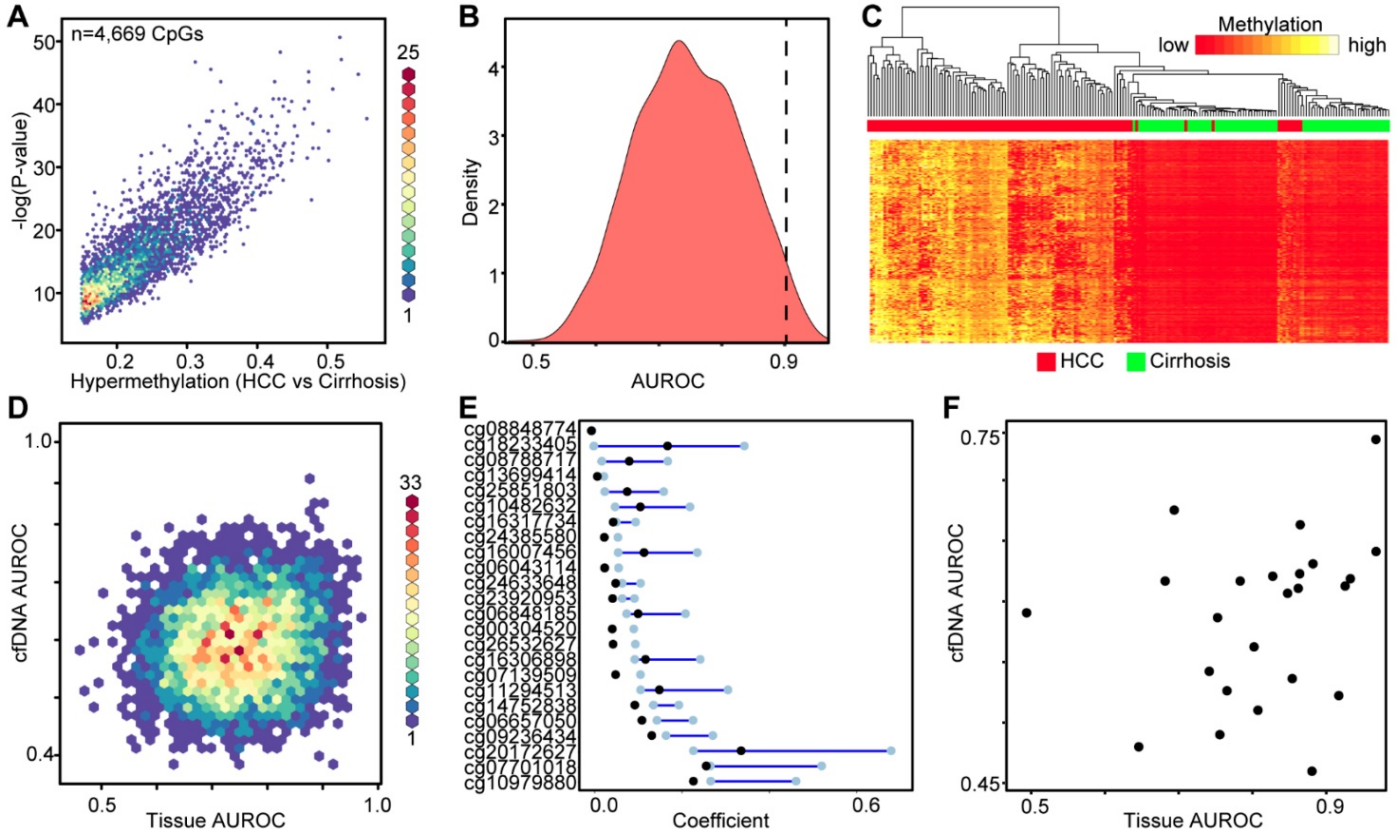
** Discovery of DNA methylation biomarkers in primary liver tissues. A)** Binned scatterplot of *de novo* DNA methylation events that significantly differ between cirrhotic only controls and HCC primary tissues (Δβ > 0.15, P < 0.05). A color bar representing density of CpGs is shown at the right. **B)** Area under the receiver operating characteristic curves for CpGs identified in (A). **C)** Heatmap of the 2,000 most differentially methylated CpGs between cirrhosis (green) and HCC (red) tissue. A color bar is shown with low methylation in red and high methylation in yellow. Red/green bar indicates tissue disease state. **D)** Binned scatterplot of AUROCs for CpGs identified in (A) in primary tissue set 1 analyzed using methylation values at the same CpG sites measured in cfDNA. **E)** Lasso regression analysis of CpGs identified in (A) presented as a dumbbell chart demarcated by the lower and upper 95% confidence interval of the coefficient (blue bar), with the original coefficient value shown as a black dot for 24 CpGs that reached the coefficient threshold of lower 95% CI > 0. **F)** A scatterplot of AUROCs from the 24 CpGs selected in (E) in cfDNA and primary tissue. The y-axis is the AUROC in cfDNA, the x-axis is the AUROC in primary tissue set 1.

Using the large public repository of 450k-derived 5mC data, including TCGA, we extracted data for 191 patient liver samples (82 cirrhosis only controls, 109 HCC with cirrhotic background, Table S1), to identify differentially methylated CpGs between disease states. We initially focused on hypermethylation events, identifying more than 4,000 CpGs that are significantly hypermethylated in HCC relative to cirrhosis-only tissues (Figure 3A). Area under the receiver operating characteristic curve (AUROC) analysis shows that a substantial proportion of these CpGs robustly distinguish between the two disease states (AUROC>0.9, n=145, Figure 3B). Moreover, supervised hierarchical clustering of the top 2,000 most differentially methylated CpGs confirms that the two disease states are significantly different (Chi-square test, p=5.81E-30, Figure 3C). Plotting the AUROCs for the highest performing CpG sites derived from primary tissue, against the AUROCs derived from the same CpGs in data derived from our cfDNA methylation dataset, reveals that tissue-derived CpGs perform much more robustly in primary tissue than in surrogate plasma cfDNA (Figure 3D). Indeed, very few tissue-derived CpGs had AUROCs above 0.75 in the cfDNA-derived methylation dataset (n=83), while significantly more reached the same threshold (AUROC > 0.75) in primary tissue (n=2,168). To further refine CpGs that distinguish between the two disease states, we performed Lasso regression analysis on the 4,669 differentially methylated CpGs identified in the primary tissue-based analysis (Figure 3E). To account for potential bias in the data, we performed 100 bootstraps and calculated the 95% confidence interval for each coefficient. We filtered the CpGs most likely to have weight by removing all CpGs with a lower bound confidence interval less than or equal to zero. Twenty four CpGs demonstrate lower bound 95% confidence intervals above zero, and were selected for further analysis. Interestingly, markers with the most weight in primary tissue are unable to distinguish between cirrhosis only control and HCC disease states using 5mC data derived from cfDNA (using a threshold of 0.75, Figure 3F). As individual markers identified from primary tissue are incapable of separating non-tumor from tumor in cfDNA, we examined whether a specific combination of the post-regression CpGs could form a panel to accurately diagnose HCC. Using a recursive partitioning-based approach, we stepwise added individual CpGs to create panels of between 1-10 CpGs using the cfDNA data on CpGs originally identified in primary tissue. While adding additional markers improves performance of cfDNA-derived panels, the AUROC plateaus at 0.9091 with a nine-marker panel (Figure S3). On the other hand, in the original tissue dataset even a single marker segregates non-tumor from tumor at an AUROC > 0.95. Moreover, an independent primary tissue derived dataset (tissue set 2) rapidly reaches an AUROC of 0.95 with as few as three markers irrespective of cirrhosis status, validating the original finding from primary tissue. Importantly, this independent primary tissue dataset shows similar global differences in methylation patterns as for tissue set 1, even though the cirrhosis status of tissue set 2 is ambiguous (Figure 2 bottom panels compared to Figures S4A-C). Altogether, these findings demonstrate that 5mC data derived from primary tissue yields differentially methylated CpGs that robustly distinguish between non-tumor and tumor tissue, but these same markers do not perform nearly as well in 5mC data derived directly from cfDNA. This finding further suggests that cfDNA 'contamination' from other cell types in blood, including leukocytes and cirrhotic tissue-derived cells in HCC patients, could hinder validation of DNA methylation biomarkers in cfDNA discovered using primary tissue.

### Genome-wide discovery of 5mC markers directly from cfDNA

**Figure 4:**
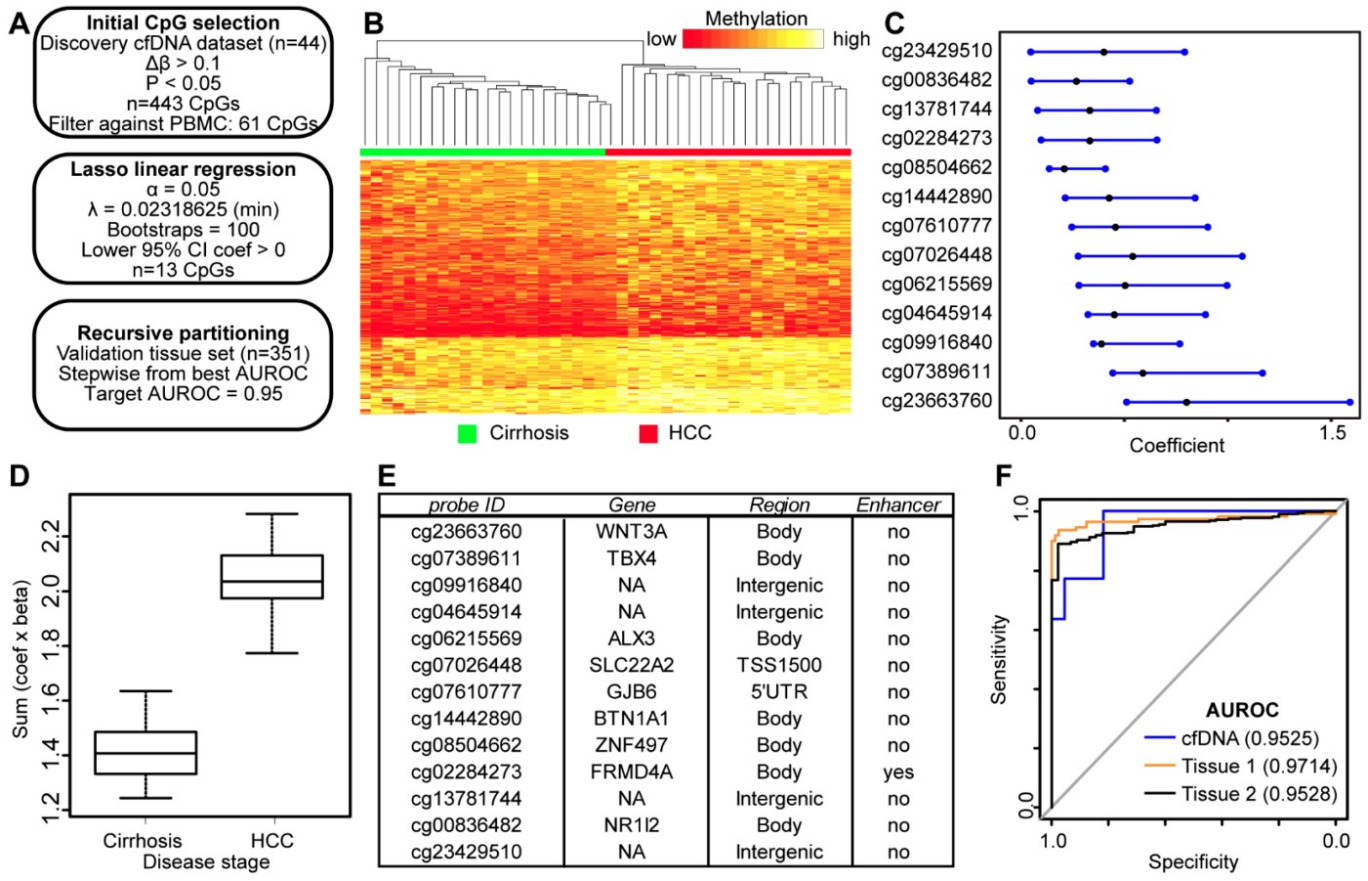
** Discovery and validation of DNA methylation biomarkers derived from cfDNA. A)** Schematic representation of the analytic process used to identify biomarkers directly from genome-wide cfDNA methylation data. **B**) Heatmap of 443 CpGs showing differential methylation between cirrhotic only control and HCC patient-derived cfDNA (Δβ > 0.1, P < 0.05). A color bar is shown to indicate 5mC level. **C**) Resultant 95% confidence intervals for positively-weighted coefficients identified by Lasso regression of CpGs from part (B). **D**) Boxplot of the additive sum of coefficients multiplied by β values for the 13 CpGs identified in (C) in cirrhotic- and HCC-derived cfDNA samples. **E**) List of the high performing CpGs identified from cfDNA along with their associated gene(s), genic features, and link to liver-specific enhancers. **F**) Receiver operating characteristic curves for a 5 CpG panel in cfDNA (blue), and in two independent primary tissue sets (orange, set 1; black, set 2). CpGs used: cg04645914, cg06215569, cg23663760, cg13781744, and cg07610777.

Since we failed to identify high performing DNA methylation-based biomarkers in cfDNA discovered from primary liver tissue, we made use of our genome-wide 5mC data derived directly from cfDNA to discover 5mC markers that distinguish between cancer and non-cancer states. As such, we used our 450k data from cfDNA as a discovery cohort and 5mC data from two independent primary tissue validation cohorts in an analytic pipeline similar to that used in our analysis of tissue-based 5mC markers (Figure 4A). We first selected 443 CpGs significantly hypermethylated (p<0.05, Δβ > 0.1) in HCC patient-derived cfDNA relative to cirrhosis only control patient cfDNA. These CpGs robustly separate cirrhotic and HCC patient samples by hierarchical clustering (Figure 4B). As an additional measure to account for potential 'contamination' from leukocyte DNA, we filtered these CpGs against sorted blood cell types to ensure that DNA methylation in HCC cfDNA is higher than that in leukocytes at these sites. We further filtered the data by removing CpGs hypomethylated in primary tissue. We then performed Lasso regression on the 61 remaining CpGs to select for the most discriminating markers. As described above, our pipeline filters out CpGs from bootstrap analysis with a lower bound confidence interval less than or equal to zero (Figure 4A). This yields 13 CpGs for downstream analysis predicted to have weight in our cfDNA dataset to segregate cirrhosis and HCC samples (Figure 4C). After multiplying β values by the coefficient for each CpG, our model perfectly segregates cfDNA derived from cirrhosis only control and HCC patients with background cirrhosis (Figure 4D).

To determine if these CpGs segregate non-tumor from tumor based on their methylation in tissue, we extracted data for the same 13 CpGs from tissue sets 1 and 2 for analysis (Figure 4E, first column). First, we tested the additive model on all 13 CpGs, observing a similar trend for both tissue datasets (Figure S5). As described above, we combined individual CpGs together to identify panels that discriminate between non-tumor and tumor states. We limited our model to five CpGs to avoid overfitting. Beginning with our best performing individual CpG (cg04645914), we stepwise added CpGs to create panels of 2-5 CpGs. Indeed, with a panel of 5 CpGs (cg04645914, cg06215569, cg23663760, cg13781744, and cg07610777), we obtain an AUROC for 0.9525 in cfDNA, replicated at 0.9714 in tissue set 1, and 0.9528 in tissue set 2 (Figure 4F). In addition, three other CpG panels reach AUROC values of 0.95 or greater in all three datasets (replacing cg07610777 with cg07389611, cg23429510, or cg14442890). Notably, tissue set 1 is controlled for cirrhosis; the controls are all confirmed cirrhotic and the experimental group is HCC with background cirrhosis, whereas tissue set 2 is independent of cirrhosis status, as the patients either had earlier stages of fibrosis, or data was not available (Table S1). Moreover, samples represent patients with a variety of environmental insults that precede disease initiation (e.g. hepatitis C viral infection, chronic alcohol intake), suggesting that the 5mC biomarker panels identified in cfDNA are applicable across liver disease etiology.

**Figure 5:**
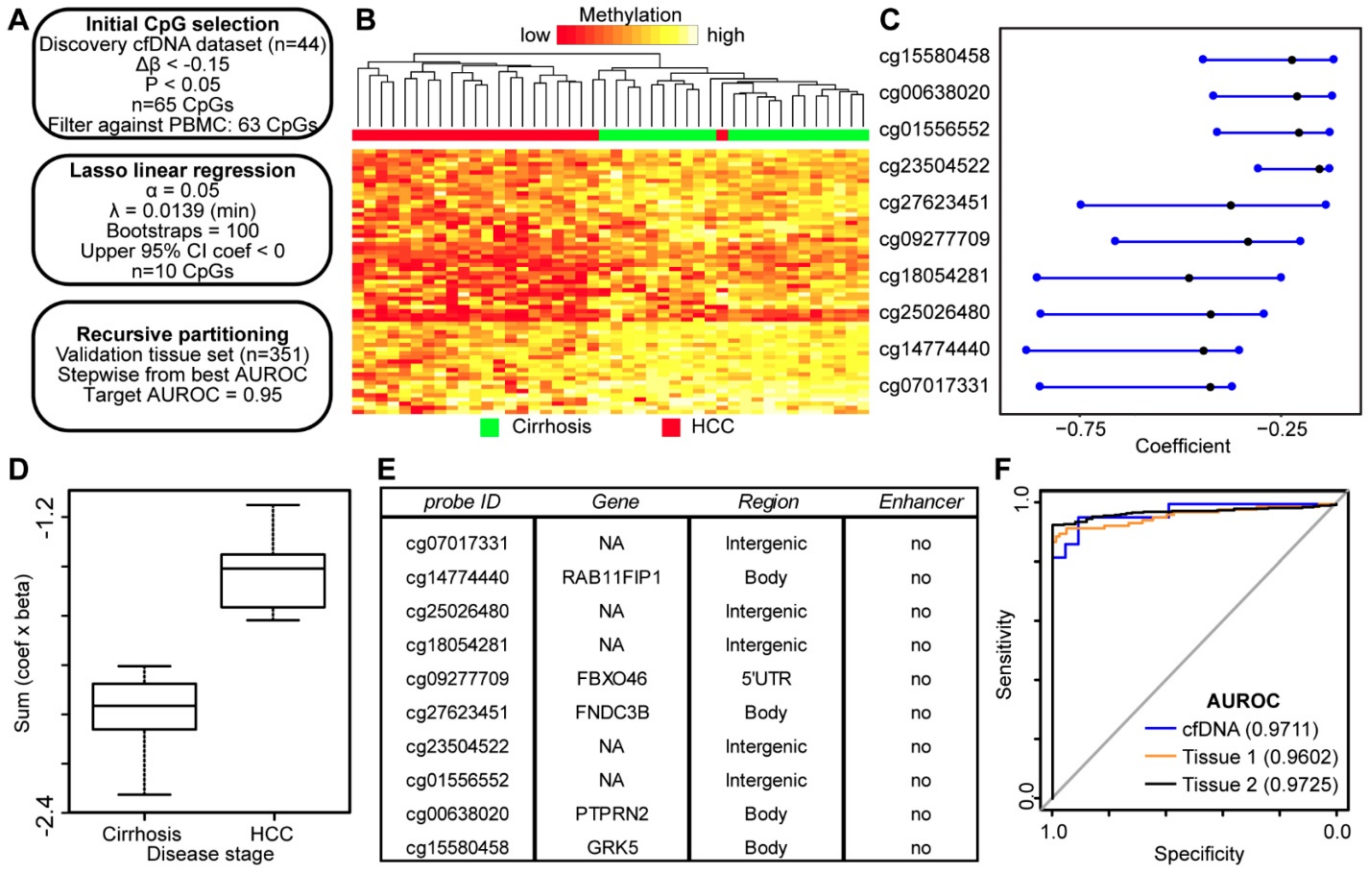
** DNA hypomethylation based markers that distinguish between non-tumor and tumor disease states. A)** Schematic representation of the analytic process used to identify hypomethylation biomarkers directly from genome-wide cfDNA 5mC datasets. **B**) Heatmap of 63 CpGs showing differential methylation between cirrhotic only control and HCC patient-derived cfDNA (Δβ <- 0.15, P < 0.05). A color bar is shown to indicate 5mC level. **C**) Resultant 95% confidence intervals for positively-weighted coefficients identified by Lasso regression of CpGs from part (B). **D**) Boxplot of the additive sum of coefficients multiplied by β values for the 10 CpGs identified in (C) in cirrhotic- and HCC-derived cfDNA samples. **E**) List of the high performing CpGs identified from cfDNA along with their associated gene(s), genic features, and link to liver-specific enhancers. **F**) Receiver operating characteristic curves for a 4 CpG panel in cfDNA (blue), and in two independent primary tissue sets (orange, set 1; black, set 2). CpGs used: cg25026480, cg14774440, cg18054281, cg00638020.

While conceptually the use of DNA hypermethylation events as biomarkers might be preferred due to the desire for a gain of 'signal' specifically in cancer-containing samples, DNA hypomethylation events have also emerged as reliable biomarkers as highlighted by Moss *et al*. using cfDNA 25, through studies of the *KCNQ1OT1* locus for imprinting disorders 41, and by several investigations of potential biomarkers for the detection of liver and other solid tumor types based on tissue 42, 43. Therefore, we performed a similar analysis as applied to hypermethylation events above, but focusing on CpGs hypomethylated in HCC relative to cirrhosis only-derived cfDNA samples (Figure 5A). Consistent with our previous data (Figure 2), we observe many more hypomethylation events (n=1,770) compared to hypermethylation events (n=444) in HCC compared to cirrhotic control cfDNA samples using equivalent cutoffs (Δβ > |0.1|, p<0.05). Since we had a greater number of significant sites, we employed a more stringent cutoff for the hypomethylation dataset (Δβ < -0.15; p<0.05), resulting in 65 differentially methylated CpGs. Filtering against normal leukocyte populations resulted in removal of only two CpGs, for a total of 63 CpGs for the Lasso regression pipeline. Importantly, these CpGs robustly segregate non-tumor from tumor samples (Fig 5B). Following linear regression, 10 CpGs remained with a discriminating coefficient (Figure 5C). As shown in Figure 5D, the model performs well in cfDNA and in both independent primary tissue datasets (Figure S6). In contrast to the hypermethylation analysis, a high proportion of CpGs from the hypomethylated sites are associated with intergenic regions (Figure 5E). With as few as four CpGs, we are able to robustly differentiate between non-tumor and tumor in cfDNA and both primary liver tissue cohorts (Figure 5F) with AUROCs above 0.95 in all three datasets (cfDNA, 0.9711; tissue set 1, 0.9602; tissue set 2, 0.9725). Taken together, this data demonstrates that both gains and losses of 5mC can be used to segregate non-tumor from tumor in HCC with and without background cirrhosis in both cell free and tissue-derived DNA.

We also employed an integrated approach where we combined the best performing hyper- and hypomethylated CpGs from our two identified panels to create a unified panel with as few CpGs as possible. Using this combined approach, we were able to generate a panel with four markers that performed well in all three data sets with AUROCs above 0.95 (Figure S7; cfDNA 0.9731, Tissue 1 0.9893, Tissue 2 0.9752). Perhaps unsurprisingly, three of the four markers in the panel are derived from the hypomethylated CpG set, which is consistent with data in Figure 5 showing that fewer CpGs are required to generate methylation panels with AUROCs greater than 0.95 when the two sets of CpGs are analyzed separately. In general, this combinatorial panel performs very well, suggesting that integrating hyper- and hypomethylated CpGs is a more efficient approach to creating biomarker panels.

Having established a panel that performed well by combining hypermethylated and hypomethylated CpG sites, we sought to validate methylation differences at these CpGs in an independent cfDNA sample set using bisulfite pyrosequencing. We obtained plasma and isolated cfDNA from 15 cirrhosis only patients and 15 cirrhosis with concurrent HCC patients from the NIH Common Fund-supported Extracellular RNA Communication program biorepository and the Mayo Clinic Hepatobiliary SPORE Biospecimen Bank. We then performed bisulfite pyrosequencing on methylated and unmethylated controls along with the 30 validation samples (Figure S8A). After bisulfite pyrosequencing, a 5-marker panel consisting of cg25026480, cg18054281, cg07610777, cg13781744, and cg04645914 produced an AUROC of 0.9556, compared to 0.996 from the discovery cohort based on Infinium 450k data (Figure S8B). Thus, differentially methylated CpGs that distinguish HCC from non-tumor patients identified in cfDNA from Infinium 450k-based analysis, and validated from independent primary tissue sources, perform well and validate in an independent plasma-derived sample set by an alternative locus-specific assay. This suggests that DNA methylation profiling directly in the surrogate tissue of interest (in this case, plasma) is a valuable approach for defining biomarker panels.

An overwhelming majority of patients that present with HCC have concurrent cirrhosis, and cirrhotic patients are advised to undergo surveillance for HCC. Since we are interested in detecting HCC at the earliest stage possible, we parsed out patients who had early-stage HCC based on TNM staging as reported by TCGA. A majority of patient samples from TCGA with methylation data available are early stage (T1; 175/377; 46%). As such we were able to calculate AUROCs from primary tissue data, demonstrating that both our hypermethylated and hypomethylated panels performed well in this patient population (Figure S9; AUROC >0.99 for both panels). This suggests that our panels are applicable for the detection of early-stage HCC.

### Functional annotation of differentially methylated biomarker panel CpGs

While the primary endpoint of this research is to identify 5mC-based biomarkers for HCC, epigenetic regulation at these candidate CpGs may also reveal biologically relevant processes. To examine the potential functional relevance of differentially methylated CpGs discovered in cfDNA, we examined the 267 genes linked to 443 significantly hypermethylated CpGs and the 930 genes linked to 1,770 significantly hypomethylated CpGs using Ingenuity Pathway Analysis (IPA) and the Genomic Regions Enrichment of Annotations Tool (GREAT) (Figure S10). The majority of identified CpGs are linked to one or two genes (Figure S10A). Hypermethylated CpGs tend to reside closer to the transcription start site (TSS), while hypomethylated CpGs are enriched in regions more distant to the TSS (relatively more hypomethylated CpGs were further than 50kb from the TSS, Figure S10B). Ontology analysis by IPA emphasizes the links between these differential methylation events and cancer, organismal injury, and gastrointestinal disease (Figure S10C). Diseases and canonical pathway enrichments are linked to liver cancer-related terms (e.g. liver carcinoma/hepatobiliary system cancer), and liver function terms (e.g. hepatic cholestasis/apelin liver signaling, Figures S10D-E) respectively, with other more general terms linked to cancer and inflammation (e.g. apoptosis, IL-3 signaling). Honing in on genes linked to the most discriminating CpGs from cfDNA (Figure 4E and Figure 5E), a similar link is observed. Indeed, *ALX3*, *GJD2*, and *SLC22A2*, are linked to the IPA pathways 'liver carcinoma' and 'hepatobiliary system cancer'. Other links include findings that WNT3A expression is frequently deregulated in HCC 44, ALX3 is differentially expressed and methylated in TCGA LIHC data 45, and expression of NR1I2 (a transcription factor that regulates *CYP3A4*, a xenobiotic metabolizing enzyme whose substrates include both sorafenib and regorafenib) is linked to sorafenib resistance in late stage HCC 46. Furthermore, demethylation of HCC cells with 5-aza-2'-deoxycytidine treatment increases NR1I2 expression 47. FNDC3B expression promotes cell migration and tumor metastases in HCC 48. To address whether there is a functional impact of these methylation changes on HCC, we examined TCGA LIHC methylation, expression, and survival data for these loci. Several of the CpGs that we identified are indeed associated with a difference in survival (e.g. cg07610777, p=0.0495). We then examined expression of the associated genes to determine if there was a relationship between transcript levels and differential methylation. For ALX3 and WNT3A, we observed hypermethylated probes in the gene body (cg06215569 and cg23663760, respectively), which corresponded with in an increase in gene expression in HCC relative to adjacent normal controls from TCGA. In a similar vein cg00638020, which is associated with the *PTPRN2* gene, demonstrated reduced expression and gene body hypomethylation in HCC relative to normal controls. Thus, these data suggest that a subset of the CpGs in our panels have a functional relationship with expression, particularly in the context of gene body methylation. Overall, many of the CpGs identified in our DNA methylation-based panels from cfDNA appear to play a role in HCC initiation and/or progression, suggesting that differential methylation of these sites is not simply a passenger event, but rather is linked to the epigenetic processes that drive HCC.

## Discussion

In the current study we performed an unbiased genome-wide survey of DNA methylation patterns directly from cfDNA in liver disease patients with/without HCC. Using several milliliters of plasma we obtained sufficient cfDNA for a genome-wide 450k-type analysis and identified from these samples a set of differentially methylated CpGs (cg04645914, cg06215569, cg23663760, cg13781744, and cg07610777) that robustly differentiate between case and control groups from both the discovery cohort (AUROC of 0.95) and two independent validation tissue-based cohorts (AUROCs of 0.97 and 0.95). These high performing sites are aberrantly methylated in HCC relative to cirrhosis and relative to cfDNA derived from normal healthy controls, suggesting that they will perform even with variable levels of cfDNA contributed from normal blood cells. Given that most cfDNA based marker identification efforts start with the primary tissue under study, we independently derived a set of differential CpG methylation events from nearly 200 primary cirrhotic and HCC tissues, which segregate these two disease states robustly (e.g. AUROC of 0.9696 for a 5-marker hypermethylation panel). When we compared the performance of the best tissue-derived methylation markers in cfDNA, they generally performed only moderately well (AUROCs around 0.88) and were inferior to those derived from direct screening of cfDNA methylation. Interestingly, many of the genes linked to these differentially methylated CpGs are differentially expressed between cirrhotic and HCC tissues or have been implicated in hepatocarcinogenesis (e.g. WNT3A, NR1I2; data not shown). Importantly, all three of our panels (hypermethylated, hypomethylated, and combined) perform better than AFP values derived from cfDNA (AUROC = 0.841) or TCGA (AUROC = 0.5525) samples. Thus, our findings suggest that 'learning' differential CpG methylation events directly from cfDNA, the ultimate non-invasive clinical test screening substrate, leads to more robust and distinct hits than those derived from primary tissue, and that our panel of DNA methylation markers are promising candidates for validation in larger patient cohorts and prospective studies in the high-risk cirrhotic liver patient population.

A growing number of studies have examined DNA methylation at single candidate gene loci derived from tissue-based studies, in cfDNA as potential cancer biomarkers. A meta-analysis of over 20 studies in HCC prior to 2015 revealed that while promising, tissue candidate gene-derived markers generally do not perform sufficiently well for clinical test development unless coupled with AFP 49. A major advantage of cfDNA methylation stems from the cell-type specificity of epigenetic marks like 5mC, permitting identification of not only a tumor or disease signature, but also the cell/tissue of origin for that aberrant signature 25. Indeed this cell-type specificity has been capitalized on as a marker of tissue damage in the context of diabetes, organ transplant (bone marrow, liver, and islets), multiple sclerosis, brain injury, sepsis, and pancreatitis, and in several cancers including hepatocellular, pancreatic, and follicular lymphoma, by quantifying perturbations to the normal cellular contributions to plasma cfDNA through computational deconvolution of differential methylation of specific cell populations 50-52. These latter studies use 5mC patterns specific to a given normal tissue as a way to quantify cell death or disease in that tissue, rather than for the identification of marks that are disease or cancer type-specific, but this use for cfDNA methylation is nonetheless extremely promising. Application of genome-wide 5mC screens, such as reduced representation bisulfite sequencing, to HCC tissues in a recent study greatly increased the pool of differentially methylated biomarkers and pinpointed a 6 CpG marker panel that performed robustly when examined in cfDNA, showing that primary diseased tissue can serve as a suitable source of cfDNA markers, particularly if coupled to large-scale screens with deep CpG coverage 39. Increasingly, however, unbiased genome-wide 5mC screens are being applied directly to cfDNA and strongly suggest that markers identified in this way have robust performance in detecting early stage cancers 53. In the context of liver disease and HCC, both methylation arrays and whole genome bisulfite sequencing have been applied directly to cfDNA, and revealed several promising markers, but these studies generally used few samples, pooled cfDNA from multiple patients, or were focused exclusively on HBV-driven liver disease 50, 54, 55. Given that hepatocytes contribute up to 10% of the plasma cfDNA in normal healthy individuals (two studies ranged from 1-10% 25, 50) and the liver is a highly vascular organ that receives nearly a quarter of the blood output from the heart 56, tumors within the liver may be particularly well suited for a cfDNA methylation-based approach to early cancer detection.

Use of a broader genome-wide approach as the output for DNA methylation biomarkers in the surrogate tissue of interest, rather than a small marker panel, is an exciting prospect and motivates two interesting future directions to query: 1) could genome-wide assays using small quantities of plasma be used diagnostically, and 2) do these CpGs (hundreds or thousands in this case) perform equally well or better than smaller 5-10 CpG marker panels? Recent research suggests that Illumina Infinium methylation arrays (both the HumanMethylation450k array and the newer generation HumanMethylationEPIC array, which profiles roughly twice the number of sites as the 450k) show promise as a test platform based on interpretation of Clinical Laboratory Improvement Amendments (CLIA) guidelines 57. This could be further strengthened if machine-learning techniques are designed to incorporate all/many probes on the array to make a single call for cases and controls. While locus-specific assays could be designed to create a panel of CpGs that distinguish between disease groups (e.g. 39), it is an exciting prospect that a genome-wide approach might one day be utilized to create an even more robust diagnostic assay, particularly as the cost of such assays decline.

While our study identified CpGs that robustly segregate non-tumor from tumor patients, additional work is needed. Future studies include validation of our findings in a larger independent cfDNA-based dataset, either at the genome-wide level or through locus-specific assays. Moreover, it has been shown that specific genes (e.g. *p16INK4A*) are hypermethylated in multiple tumor types, which could lead to “false negatives” for HCC diagnosis due to the presence of another cancer. One of our primary tissue datasets was collected independent of cirrhosis status and still validated our findings from cfDNA, suggesting that these markers will also perform well in patients without cirrhosis, but more careful examination of this point is required. While these patients would not typically be surveilled for HCC, non-cirrhotic patients with HBV and active hepatitis (among others) also represent high-risk groups that could benefit from a non-invasive screen. Future work will also be required to address the efficacy of cfDNA methylation biomarkers in a more inclusive patient cohort, especially collection of cfDNA from multiple sites and patient populations.

## Supplementary Material

Supplementary figures and tables.

## Abbreviations

DNMT: DNA methyltransferase
TET: Ten-eleven translocation
TCGA LIHC: The Cancer Genome Atlas liver hepatocellular carcinoma
450k: Illumina Infinium HumanMethylation450k BeadChip Array
AUROC: area under the receiver operating characteristic curve
5mC: 5-methylcytosine
cfDNA: cell-free DNA
