# Supplementary material for: Genome-wide discovery and validation of diagnostic DNA methylation-based biomarkers for hepatocellular cancer detection in circulating cell free DNA
Source: Theranostics. 2019 Sep 25;9(24):7239–50. doi: 10.7150/thno.35573 (PMC6831291; doi:10.7150/thno.35573)
Supplement: Supplementary file 1 — Supplementary figures and tables. [file thnov09p7239s1.pdf]

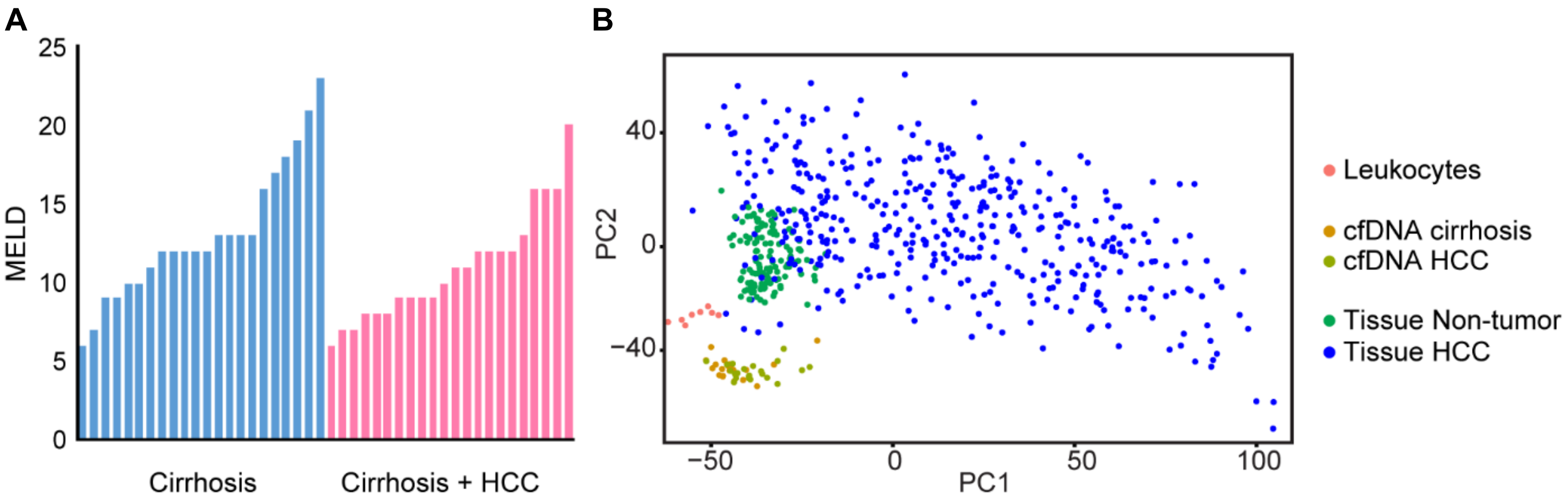

Supplemental Figure S1

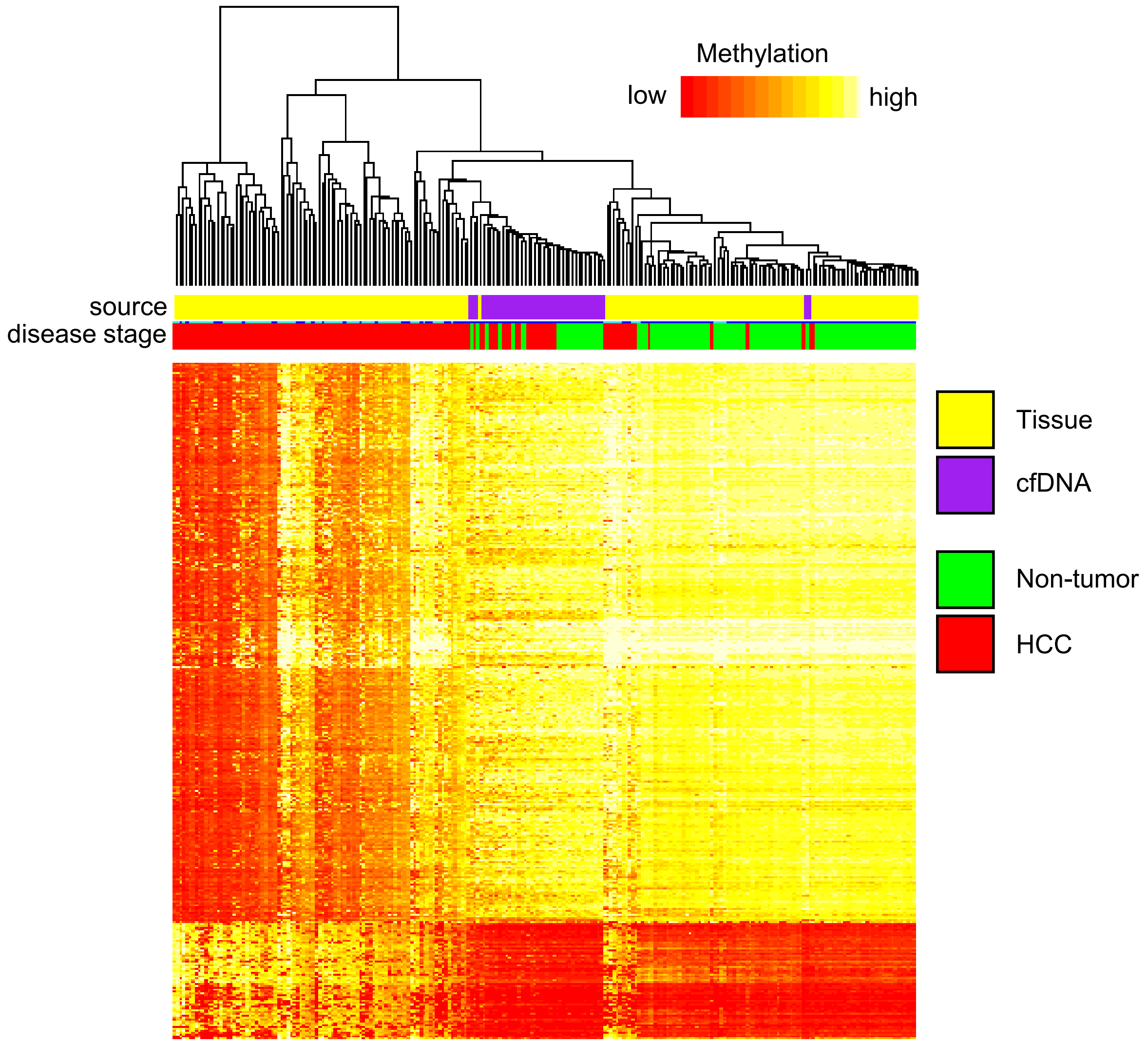

Supplemental Figure S2

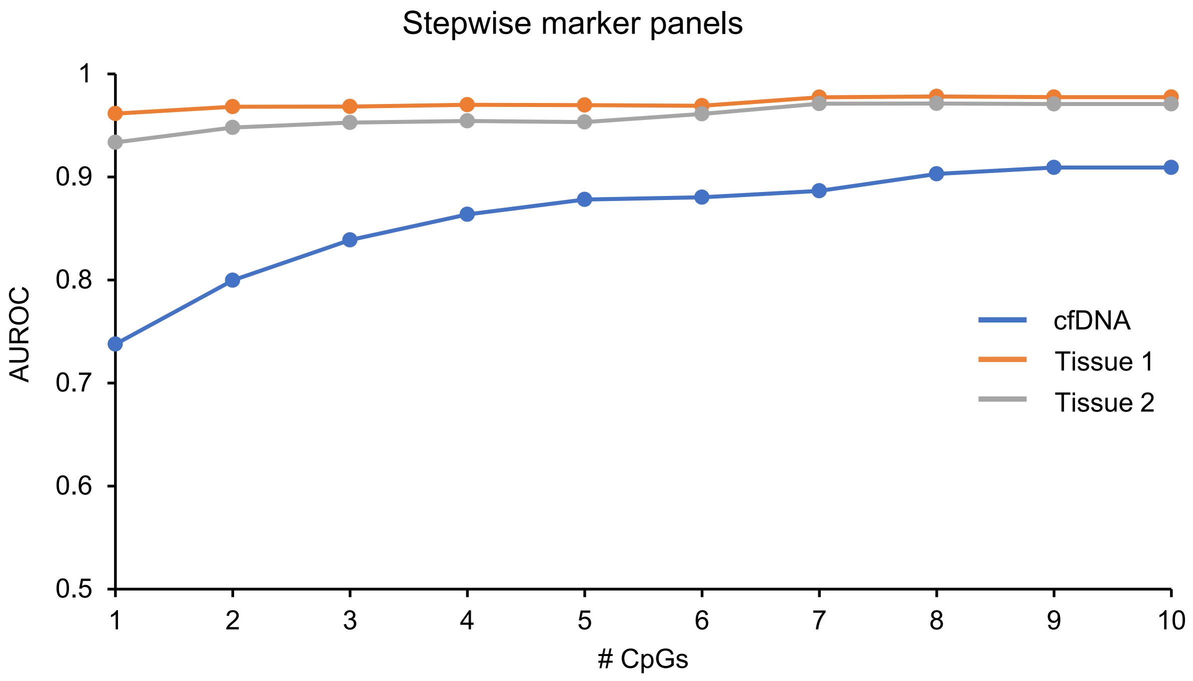

Supplemental Figure S3

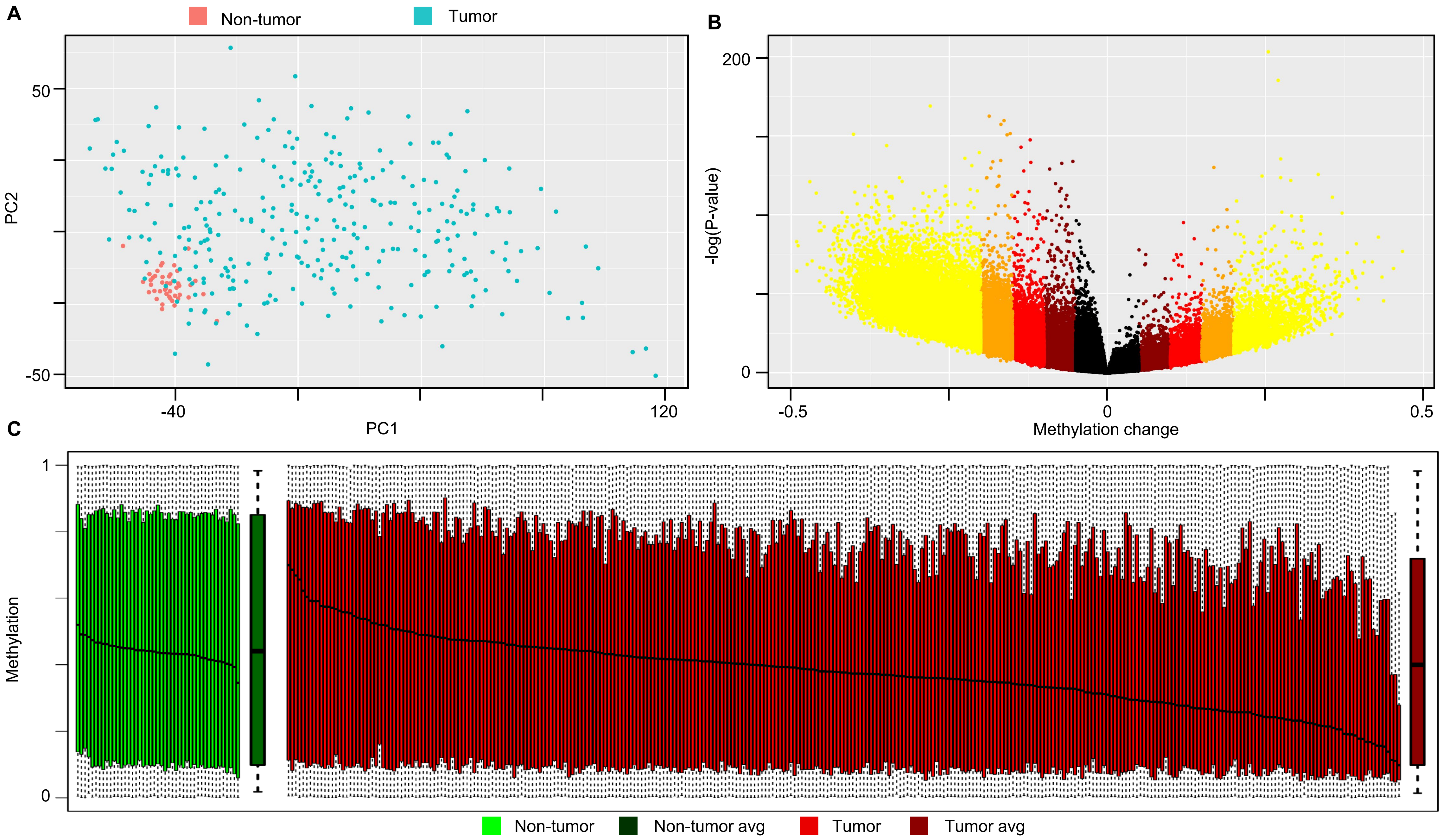

Supplemental Figure S4

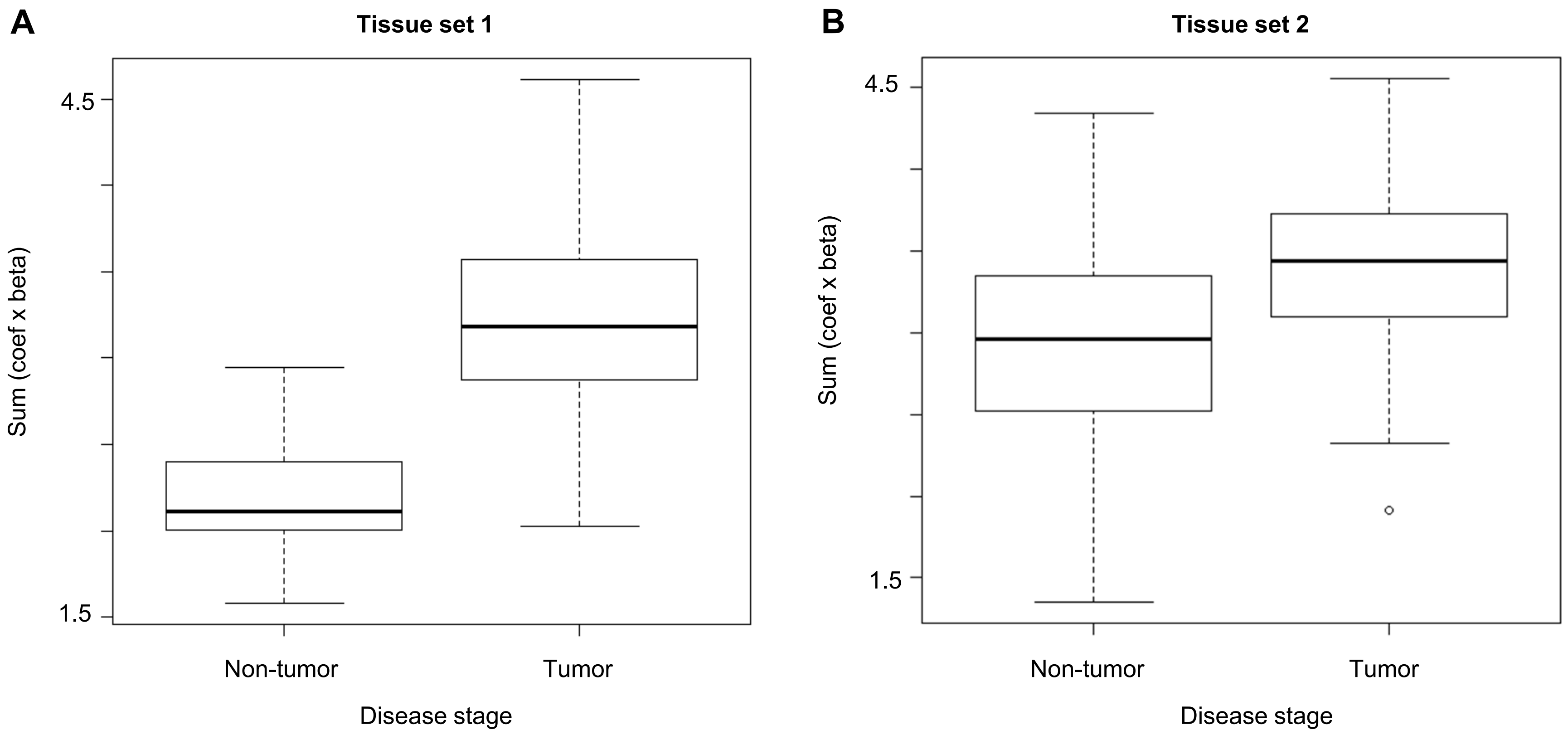

Supplemental Figure S5

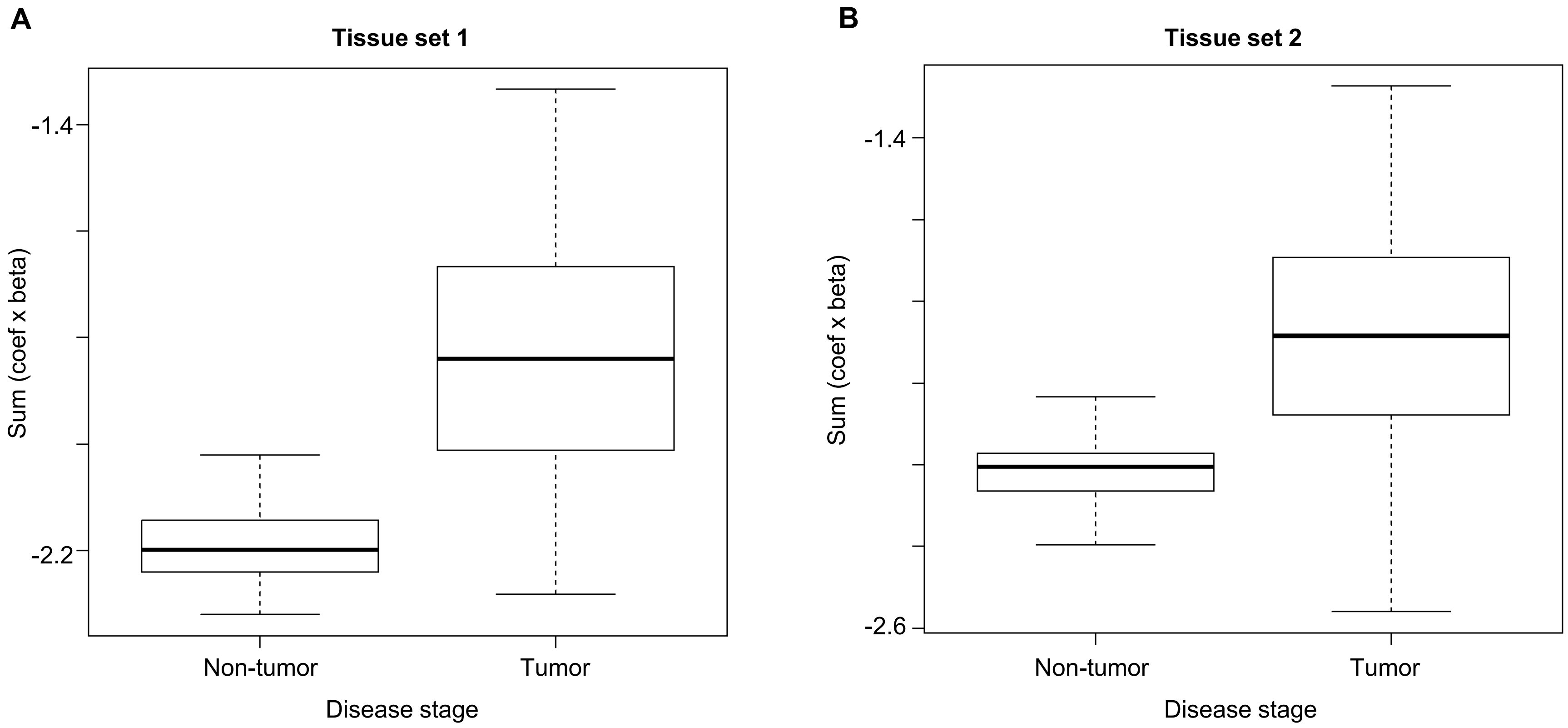

Supplemental Figure S6

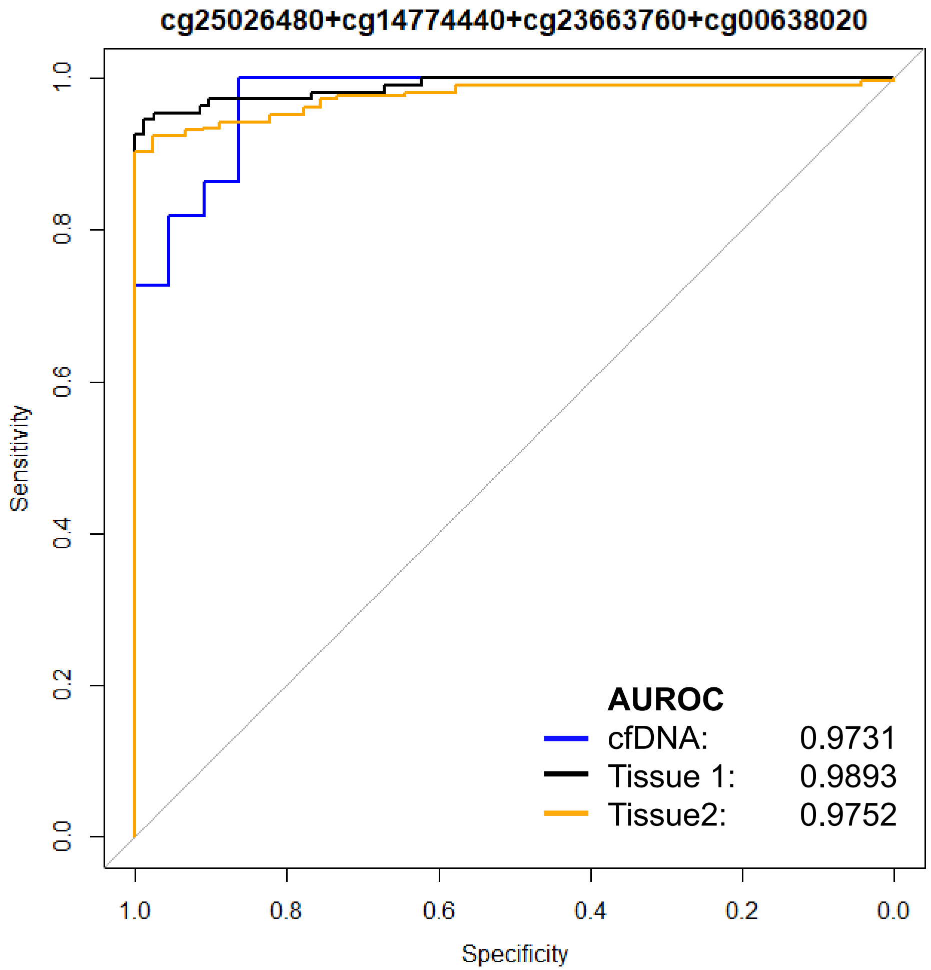

Supplemental Figure S7

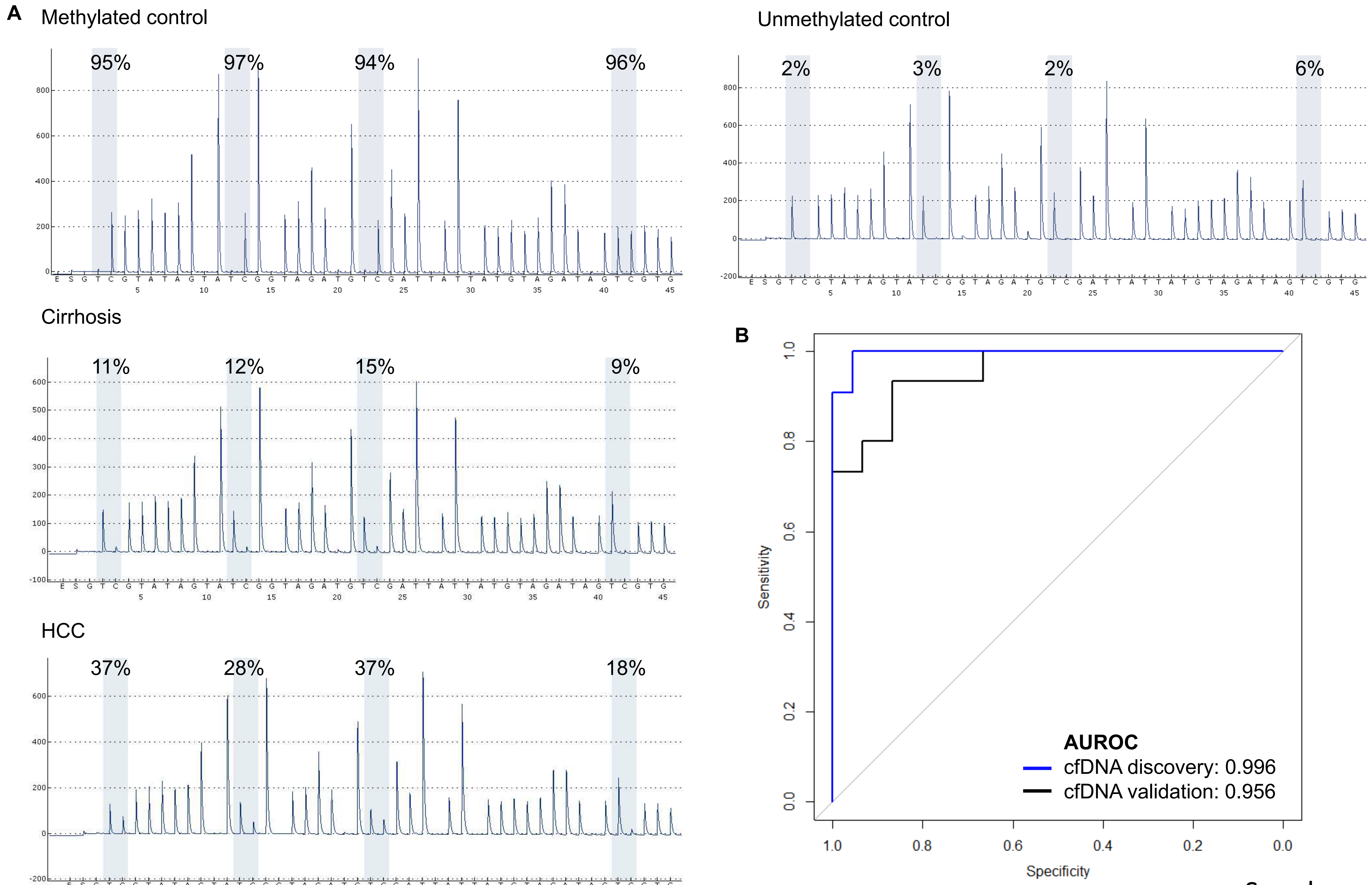

Supplemental Figure S8

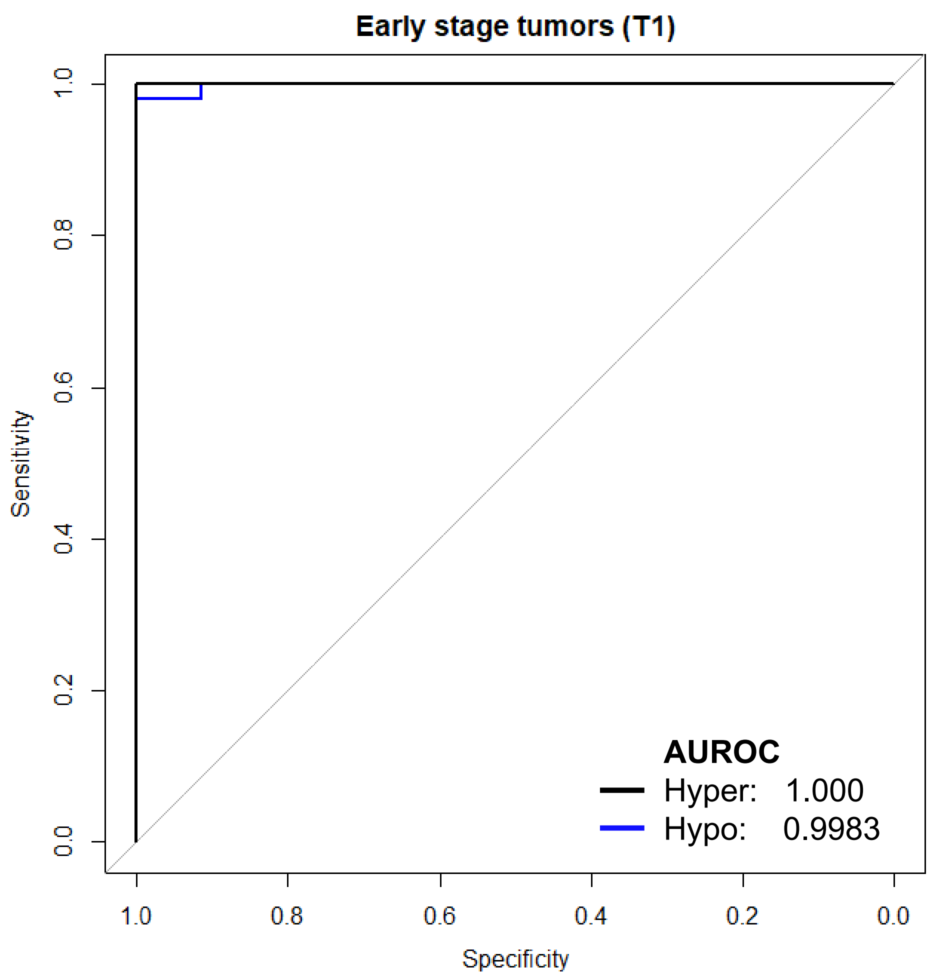

Supplemental Figure S9

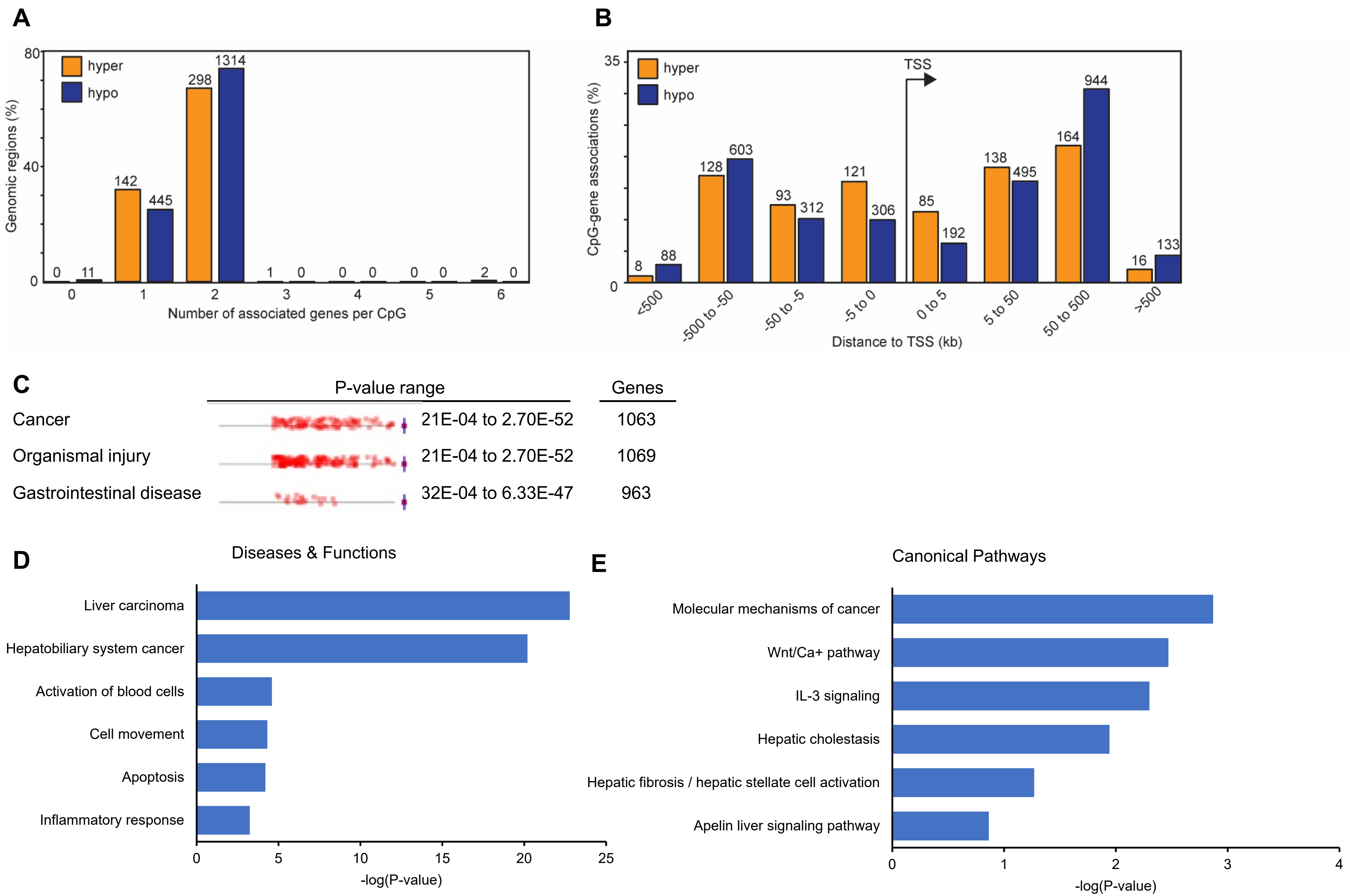

Supplemental Figure S10

Hlady *et al.*, Supplemental Material for:

Genome-wide discovery and validation of diagnostic DNA methylation-based biomarkers for hepatocellular cancer detection in circulating cell free DNA

Supplemental Figure Legends

Fig. S1: Characterization of the cfDNA cohort and its relationship to public methylation datasets. A) Bar chart depicting MELD scores for 22 cirrhosis controls (blue) and 22 cirrhosis with HCC (pink) cfDNA samples for which 450k analysis was performed. **B)** Principal component analysis using all post-quality control (QC) CpGs on the 450k array for all four datasets (cfDNA in brown/green for non-tumor/tumor respectively, normal leukocytes in peach, primary tissue sets 1 and 2 non-tumor/tumor in green/blue).

Fig S2: Unsupervised hierarchical clustering of primary tissue and cfDNA. A heatmap depicting primary tissue (yellow) and cfDNA-derived (purple) non-tumor (green) and tumor (red) DNA methylation data from the top 10,000 most variable CpGs. Primary non-tumor and tumor tissues segregate independently, while cfDNA is less distinctly segregated, likely due to the 'contamination' or mixing of DNA derived from cirrhotic portions of the liver and regions of the liver containing the HCC, for cfDNA samples derived from patients with HCC with background cirrhosis.

Fig. S3: Performance of cfDNA marker panels in CpGs discovered from primary tissue. A line chart depicting CpG marker panels ranging from 1 to 10 CpGs and the corresponding AUROC in cfDNA (blue), and in two independent tissue datasets (orange and grey for tissue

sets 1 and 2, respectively). Individual CpGs were stepwise added on to create increasingly more discriminating panels, which plateaued around 0.9 in cfDNA at 9 CpGs.

Fig. S4: Basic characterization of tissue set 2 cohort. A) Principal component analysis in n=45 non-tumor (peach) and 306 HCC (blue) tissues using all CpGs on the array after QC filtering (including removal of X, Y, and SNP-containing probes). **B)** Volcano plots of 5mC changes plotted against -log P values between non-tumor and tumor samples. Stepwise coloring of changes is based on delta beta values (0.05 increments) with black less than 0.05, dark red 0.05-0.1, red 0.1-0.15, orange 0.15-0.20, and yellow greater than 0.20. **C**) Overall methylation level bar charts (as beta value) for individual non-tumor (green), average of all non-tumor (dark green), individual tumor (red), and average of all tumor (dark red) primary tissues.

Fig S5: Performance of the hypermethylated CpG marker panel in primary tissue discovered from cfDNA. Boxplots of tissue set 1 (left) and tissue set 2 (right) representing the sum of 13 Lasso regression-derived CpGs from cfDNA methylation data.

Fig S6: Performance of the hypomethylated CpG marker panel in primary tissue discovered from cfDNA. Boxplots of tissue set 1 (left) and tissue set 2 (right) representing the sum of 10 Lasso regression-derived CpGs from cfDNA methylation data.

Fig S7: Combined hypermethylated and hypomethylated CpG panel. A receiver operating characteristic curve for a 4 CpG panel in cfDNA (blue), and in two independent primary tissue

sets (black, set 1; orange, set 2). CpGs used: cg2502648, cg14774440, cg23663760, cg00638020.

Fig S8: Validation of a 5-marker CpG panel in an independent cfDNA cohort.

A) Pyrograms derived from fully methylated and unmethylated control DNAs for the cg07610777-containing amplicon (top two panels). Representative examples of methylation values obtained from cirrhosis only and cirrhosis with HCC for this region derived from cfDNA (lower left two panels). **B)** Using 15 cirrhosis only and 15 cirrhosis with HCC samples, we preformed bisulfite pyrosequencing on amplicons surrounding cg18054281, cg25026480, cg07610777, cg13781744, and cg04645914. The combined AUROC from discovery cfDNA 450k data was 0.996, and the validation by bisulfite pyrosequencing in 30 independent samples was 0.956.

Fig S9: Combined hypermethylated and hypomethylated CpG panel in early stage HCC.

A receiver operating characteristic curve for a hypermethylated (black; n=5 CpGs) and hypomethylated (blue; n=4 CpGs) CpG panels in early stage (T1) HCC using data derived from TCGA. The CpG marker panels used for this analysis are derived from Figure 4 (hypermethylated) and Figure 5 (hypomethylated).

Fig S10: Differentially methylated CpGs from cfDNA are linked to liver cancer-related processes. A) Bar chart depicting the number of genes associated with each CpG. The majority of both hyper- (orange) and hypo-methylated (blue) CpGs are associated with 1-2 genes. **B)** The location of the same set of CpGs relative to the transcription start site (TSS) of the associated genes. **C)** An IPA summary of the categories, P-value ranges, and number of

genes associated with the overarching categories ‘cancer’, ‘organismal injury’, and ‘gastrointestinal disease’ linked to differentially methylated CpGs. Bar charts of diseases/functions **(D)**, and canonical pathways **(E)** from IPA for genes associated with differentially methylated CpGs.

Table S1: Clinical information for patient samples used in this study.

Tube_ID	Source	Ishak score	HCC	MELD	AFP	Yield (ng/mL)
Mayo_cfDNA-1	cfDNA	6 - Established Cirrhosis	no	18	5.6	102.84
Mayo_cfDNA-10	cfDNA	6 - Established Cirrhosis	no	13	5.5	9.45
Mayo_cfDNA-11	cfDNA	6 - Established Cirrhosis	no	12	3.2	9.68
Mayo_cfDNA-12	cfDNA	6 - Established Cirrhosis	no	7	0.8	14.50
Mayo_cfDNA-13	cfDNA	6 - Established Cirrhosis	no	12	1.9	11.31
Mayo_cfDNA-14	cfDNA	6 - Established Cirrhosis	no	12	2	13.71
Mayo_cfDNA-15	cfDNA	6 - Established Cirrhosis	no	21	2.2	9.09
Mayo_cfDNA-16	cfDNA	6 - Established Cirrhosis	no	11	3.4	42.38
Mayo_cfDNA-17	cfDNA	6 - Established Cirrhosis	no	13	2.9	21.30
Mayo_cfDNA-18	cfDNA	6 - Established Cirrhosis	no	23	4.6	26.16
Mayo_cfDNA-19	cfDNA	6 - Established Cirrhosis	no	6	0.8	84.96
Mayo_cfDNA-2	cfDNA	6 - Established Cirrhosis	no	10	2.3	16.56
Mayo_cfDNA-21	cfDNA	6 - Established Cirrhosis	no	10	N/A	9.93
Mayo_cfDNA-22	cfDNA	6 - Established Cirrhosis	no	12	1.7	11.46
Mayo_cfDNA-23	cfDNA	6 - Established Cirrhosis	no	12	N/A	9.68
Mayo_cfDNA-24	cfDNA	6 - Established Cirrhosis	no	9	3.2	9.56
Mayo_cfDNA-25	cfDNA	6 - Established Cirrhosis	no	9	N/A	32.64
Mayo_cfDNA-27	cfDNA	6 - Established Cirrhosis	yes	16	6949	12.61
Mayo_cfDNA-29	cfDNA	6 - Established Cirrhosis	yes	8	42400	208.08
Mayo_cfDNA-3	cfDNA	6 - Established Cirrhosis	no	19	4.8	16.59
Mayo_cfDNA-30	cfDNA	6 - Established Cirrhosis	yes	9	107	9.45
Mayo_cfDNA-33	cfDNA	6 - Established Cirrhosis	yes	12	303	13.66
Mayo_cfDNA-34	cfDNA	6 - Established Cirrhosis	yes	10	1495	10.19
Mayo_cfDNA-37	cfDNA	6 - Established Cirrhosis	yes	8	21	10.89
Mayo_cfDNA-38	cfDNA	6 - Established Cirrhosis	yes	6	3	18.73
Mayo_cfDNA-39	cfDNA	6 - Established Cirrhosis	yes	11	66	10.72
Mayo_cfDNA-40	cfDNA	6 - Established Cirrhosis	yes	9	3.7	19.05
Mayo_cfDNA-42	cfDNA	6 - Established Cirrhosis	yes	12	9312	8.10
Mayo_cfDNA-43	cfDNA	6 - Established Cirrhosis	yes	13	7.1	17.41
Mayo_cfDNA-46	cfDNA	6 - Established Cirrhosis	yes	9	6.8	21.64
Mayo_cfDNA-48	cfDNA	6 - Established Cirrhosis	yes	7	6.8	32.48
Mayo_cfDNA-53	cfDNA	6 - Established Cirrhosis	yes	11	2787	9.34
Mayo_cfDNA-54	cfDNA	6 - Established Cirrhosis	yes	16	171	9.71
Mayo_cfDNA-55	cfDNA	6 - Established Cirrhosis	yes	20	3.8	8.83
Mayo_cfDNA-56	cfDNA	6 - Established Cirrhosis	yes	9	5.2	8.92
Mayo_cfDNA-58	cfDNA	6 - Established Cirrhosis	yes	12	42348	27.31
Mayo_cfDNA-59	cfDNA	6 - Established Cirrhosis	yes	8	12	20.48
Mayo_cfDNA-6	cfDNA	6 - Established Cirrhosis	no	16	8.6	49.04
Mayo_cfDNA-62	cfDNA	6 - Established Cirrhosis	yes	16	270	34.28
Mayo_cfDNA-65	cfDNA	6 - Established Cirrhosis	yes	7	104	12.39
Mayo_cfDNA-67	cfDNA	6 - Established Cirrhosis	yes	12	17	13.32
Mayo_cfDNA-7	cfDNA	6 - Established Cirrhosis	no	13	4.3	11.74
Mayo_cfDNA-8	cfDNA	6 - Established Cirrhosis	no	17	6	12.47
Mayo_cfDNA-9	cfDNA	6 - Established Cirrhosis	no	13	2.9	18.03
Cbil-100	Tissue	6 - Established Cirrhosis	no	N/A	N/A	N/A
Cbil-165	Tissue	6 - Established Cirrhosis	no	N/A	N/A	N/A
CC-109	Tissue	6 - Established Cirrhosis	no	N/A	N/A	N/A
CC-144	Tissue	6 - Established Cirrhosis	no	N/A	N/A	N/A
CC-4	Tissue	6 - Established Cirrhosis	no	N/A	N/A	N/A
CG-197	Tissue	6 - Established Cirrhosis	no	N/A	N/A	N/A
CG-512	Tissue	6 - Established Cirrhosis	no	N/A	N/A	N/A
CG-518	Tissue	6 - Established Cirrhosis	no	N/A	N/A	N/A
CG-563N	Tissue	6 - Established Cirrhosis	no	N/A	N/A	N/A
CI-131	Tissue	6 - Established Cirrhosis	no	N/A	N/A	N/A
CI-460	Tissue	6 - Established Cirrhosis	no	N/A	N/A	N/A
CirrEtOH-110N	Tissue	6 - Established Cirrhosis	no	N/A	N/A	N/A
CirrEtOH-12	Tissue	6 - Established Cirrhosis	no	N/A	N/A	N/A
CirrEtOH-133	Tissue	6 - Established Cirrhosis	no	N/A	N/A	N/A
CirrEtOH-163	Tissue	6 - Established Cirrhosis	no	N/A	N/A	N/A
CirrEtOH-167	Tissue	6 - Established Cirrhosis	no	N/A	N/A	N/A
CirrEtOH-175	Tissue	6 - Established Cirrhosis	no	N/A	N/A	N/A
CirrEtOH-180	Tissue	6 - Established Cirrhosis	no	N/A	N/A	N/A
CirrEtOH-190	Tissue	6 - Established Cirrhosis	no	N/A	N/A	N/A
CirrEtOH-191	Tissue	6 - Established Cirrhosis	no	N/A	N/A	N/A
CirrEtOH-380	Tissue	6 - Established Cirrhosis	no	N/A	N/A	N/A
CirrEtOH-415N	Tissue	6 - Established Cirrhosis	no	N/A	N/A	N/A
CirrEtOH-444N	Tissue	6 - Established Cirrhosis	no	N/A	N/A	N/A
CirrEtOH-466N	Tissue	6 - Established Cirrhosis	no	N/A	N/A	N/A
CirrEtOH-515N	Tissue	6 - Established Cirrhosis	no	N/A	N/A	N/A
CirrEtOH-562N	Tissue	6 - Established Cirrhosis	no	N/A	N/A	N/A
CirrEtOH-57	Tissue	6 - Established Cirrhosis	no	N/A	N/A	N/A
CirrEtOH-649N	Tissue	6 - Established Cirrhosis	no	N/A	N/A	N/A
CirrEtOH-665N	Tissue	6 - Established Cirrhosis	no	N/A	N/A	N/A
CirrEtOH-674N	Tissue	6 - Established Cirrhosis	no	N/A	N/A	N/A
CirrEtOH-9	Tissue	6 - Established Cirrhosis	no	N/A	N/A	N/A
CirrHBV-120N	Tissue	6 - Established Cirrhosis	no	N/A	N/A	N/A
CirrHBV-208	Tissue	6 - Established Cirrhosis	no	N/A	N/A	N/A
CirrHBV-456	Tissue	6 - Established Cirrhosis	no	N/A	N/A	N/A
CirrHBV-5	Tissue	6 - Established Cirrhosis	no	N/A	N/A	N/A
CirrHBV-532N	Tissue	6 - Established Cirrhosis	no	N/A	N/A	N/A
CirrHBV-542N	Tissue	6 - Established Cirrhosis	no	N/A	N/A	N/A
CirrHCV-103	Tissue	6 - Established Cirrhosis	no	N/A	N/A	N/A
CirrHCV-105	Tissue	6 - Established Cirrhosis	no	N/A	N/A	N/A
CirrHCV-130	Tissue	6 - Established Cirrhosis	no	N/A	N/A	N/A
CirrHCV-153	Tissue	6 - Established Cirrhosis	no	N/A	N/A	N/A
CirrHCV-156	Tissue	6 - Established Cirrhosis	no	N/A	N/A	N/A

Tube_ID	Source	Ishak score	HCC	MELD	AFP	Yield (ng/mL)
TCGA-DD-A1EE-11	Tissue	6 - Established Cirrhosis	no	N/A	3	N/A
TCGA-DD-A1EF-01	Tissue	3,4 - Fibrous Speta	yes	N/A	16211	N/A
TCGA-DD-A1EF-11	Tissue	3,4 - Fibrous Speta	no	N/A	16211	N/A
TCGA-DD-A1EG-01	Tissue	1,2 - Portal Fibrosis	yes	N/A	16	N/A
TCGA-DD-A1EG-11	Tissue	1,2 - Portal Fibrosis	no	N/A	16	N/A
TCGA-DD-A1EH-01	Tissue	3,4 - Fibrous Speta	yes	N/A	94340	N/A
TCGA-DD-A1EH-11	Tissue	3,4 - Fibrous Speta	no	N/A	94340	N/A
TCGA-DD-A1EI-01	Tissue	6 - Established Cirrhosis	yes	N/A	24	N/A
TCGA-DD-A1EI-11	Tissue	6 - Established Cirrhosis	no	N/A	24	N/A
TCGA-DD-A1EJ-01	Tissue	0 - No Fibrosis	yes	N/A	18840	N/A
TCGA-DD-A1EJ-11	Tissue	0 - No Fibrosis	no	N/A	18840	N/A
TCGA-DD-A1EK-01	Tissue	0 - No Fibrosis	yes	N/A	19	N/A
TCGA-DD-A1EL-01	Tissue	0 - No Fibrosis	yes	N/A	5	N/A
TCGA-DD-A1EL-11	Tissue	0 - No Fibrosis	no	N/A	5	N/A
TCGA-DD-A39V-01	Tissue	0 - No Fibrosis	yes	N/A	NA	N/A
TCGA-DD-A39V-11	Tissue	0 - No Fibrosis	no	N/A	NA	N/A
TCGA-DD-A39W-01	Tissue	NA	yes	N/A	NA	N/A
TCGA-DD-A39W-11	Tissue	NA	no	N/A	NA	N/A
TCGA-DD-A39X-01	Tissue	0 - No Fibrosis	yes	N/A	10	N/A
TCGA-DD-A39X-11	Tissue	0 - No Fibrosis	no	N/A	10	N/A
TCGA-DD-A39Y-01	Tissue	0 - No Fibrosis	yes	N/A	5600	N/A
TCGA-DD-A39Z-01	Tissue	NA	yes	N/A	NA	N/A
TCGA-DD-A39Z-11	Tissue	NA	no	N/A	NA	N/A
TCGA-DD-A3A0-01	Tissue	0 - No Fibrosis	yes	N/A	1	N/A
TCGA-DD-A3A1-01	Tissue	0 - No Fibrosis	yes	N/A	6	N/A
TCGA-DD-A3A1-11	Tissue	0 - No Fibrosis	no	N/A	6	N/A
TCGA-DD-A3A2-01	Tissue	0 - No Fibrosis	yes	N/A	NA	N/A
TCGA-DD-A3A2-11	Tissue	0 - No Fibrosis	no	N/A	NA	N/A
TCGA-DD-A3A3-01	Tissue	0 - No Fibrosis	yes	N/A	6	N/A
TCGA-DD-A3A3-11	Tissue	0 - No Fibrosis	no	N/A	6	N/A
TCGA-DD-A3A4-01	Tissue	0 - No Fibrosis	yes	N/A	NA	N/A
TCGA-DD-A3A5-01	Tissue	0 - No Fibrosis	yes	N/A	55	N/A
TCGA-DD-A3A6-01	Tissue	0 - No Fibrosis	yes	N/A	NA	N/A
TCGA-DD-A3A7-01	Tissue	0 - No Fibrosis	yes	N/A	120	N/A
TCGA-DD-A3A8-01	Tissue	0 - No Fibrosis	yes	N/A	4	N/A
TCGA-DD-A3A9-01	Tissue	0 - No Fibrosis	yes	N/A	19930	N/A
TCGA-DD-A1EB-01	Tissue	0 - No Fibrosis	yes	N/A	7	N/A
TCGA-DD-A4NB-01	Tissue	NA	yes	N/A	3	N/A
TCGA-DD-A4ND-01	Tissue	0 - No Fibrosis	yes	N/A	NA	N/A
TCGA-DD-A4NE-01	Tissue	0 - No Fibrosis	yes	N/A	28	N/A
TCGA-DD-A4NF-01	Tissue	6 - Established Cirrhosis	yes	N/A	6	N/A
TCGA-DD-A4NG-01	Tissue	NA	yes	N/A	3	N/A
TCGA-DD-A4NH-01	Tissue	1,2 - Portal Fibrosis	yes	N/A	92889	N/A
TCGA-DD-A4NI-01	Tissue	0 - No Fibrosis	yes	N/A	6	N/A
TCGA-DD-A4NJ-01	Tissue	1,2 - Portal Fibrosis	yes	N/A	5	N/A
TCGA-DD-A4NK-01	Tissue	0 - No Fibrosis	yes	N/A	3	N/A
TCGA-DD-A4NL-01	Tissue	0 - No Fibrosis	yes	N/A	2	N/A
TCGA-DD-A4NN-01	Tissue	0 - No Fibrosis	yes	N/A	4223	N/A
TCGA-DD-A4NO-01	Tissue	0 - No Fibrosis	yes	N/A	3	N/A
TCGA-DD-A4NP-01	Tissue	0 - No Fibrosis	yes	N/A	2	N/A
TCGA-DD-A4NQ-01	Tissue	NA	yes	N/A	141	N/A
TCGA-DD-A4NR-01	Tissue	6 - Established Cirrhosis	yes	N/A	40250	N/A
TCGA-DD-A4NS-01	Tissue	0 - No Fibrosis	yes	N/A	2	N/A
TCGA-DD-A4NV-01	Tissue	0 - No Fibrosis	yes	N/A	3	N/A
TCGA-DD-A73A-01	Tissue	3,4 - Fibrous Speta	yes	N/A	4	N/A
TCGA-DD-A73B-01	Tissue	6 - Established Cirrhosis	yes	N/A	30	N/A
TCGA-DD-A73C-01	Tissue	0 - No Fibrosis	yes	N/A	3	N/A
TCGA-DD-A73D-01	Tissue	6 - Established Cirrhosis	yes	N/A	4	N/A
TCGA-DD-A73E-01	Tissue	0 - No Fibrosis	yes	N/A	1	N/A
TCGA-DD-A73F-01	Tissue	0 - No Fibrosis	yes	N/A	283	N/A
TCGA-DD-A73G-01	Tissue	0 - No Fibrosis	yes	N/A	2035400	N/A
TCGA-DD-AA3A-01	Tissue	0 - No Fibrosis	yes	N/A	1	N/A
TCGA-DD-AAC8-01	Tissue	1,2 - Portal Fibrosis	yes	N/A	1	N/A
TCGA-DD-AAC9-01	Tissue	6 - Established Cirrhosis	yes	N/A	5	N/A
TCGA-DD-AACA-01	Tissue	6 - Established Cirrhosis	yes	N/A	7	N/A
TCGA-DD-AACA-02	Tissue	6 - Established Cirrhosis	yes	N/A	7	N/A
TCGA-DD-AACB-01	Tissue	6 - Established Cirrhosis	yes	N/A	1902	N/A
TCGA-DD-AACC-01	Tissue	6 - Established Cirrhosis	yes	N/A	24	N/A
TCGA-DD-AACD-01	Tissue	1,2 - Portal Fibrosis	yes	N/A	2	N/A
TCGA-DD-AACE-01	Tissue	6 - Established Cirrhosis	yes	N/A	2	N/A
TCGA-DD-AACF-01	Tissue	6 - Established Cirrhosis	yes	N/A	16	N/A
TCGA-DD-AACG-01	Tissue	6 - Established Cirrhosis	yes	N/A	11718	N/A
TCGA-DD-AACH-01	Tissue	6 - Established Cirrhosis	yes	N/A	7	N/A
TCGA-DD-A1EB-11	Tissue	0 - No Fibrosis	no	N/A	7	N/A
TCGA-DD-AACJ-01	Tissue	NA	yes	N/A	25	N/A
TCGA-DD-AACK-01	Tissue	6 - Established Cirrhosis	yes	N/A	5	N/A
TCGA-DD-AACL-01	Tissue	NA	yes	N/A	1368	N/A
TCGA-DD-AACM-01	Tissue	3,4 - Fibrous Speta	yes	N/A	2	N/A
TCGA-DD-AACN-01	Tissue	NA	yes	N/A	699	N/A
TCGA-DD-AACO-01	Tissue	6 - Established Cirrhosis	yes	N/A	5	N/A
TCGA-DD-AACP-01	Tissue	NA	yes	N/A	227	N/A
TCGA-DD-AACQ-01	Tissue	NA	yes	N/A	7	N/A
TCGA-DD-AACS-01	Tissue	NA	yes	N/A	1	N/A
TCGA-DD-AACT-01	Tissue	NA	yes	N/A	4	N/A
TCGA-DD-AACU-01	Tissue	NA	yes	N/A	22	N/A
TCGA-DD-AACV-01	Tissue	NA	yes	N/A	9	N/A

CirrHCV-161	Tissue	6 - Established Cirrhosis	no	N/A	N/A	N/A
CirrHCV-164	Tissue	6 - Established Cirrhosis	no	N/A	N/A	N/A
CirrHCV-174	Tissue	6 - Established Cirrhosis	no	N/A	N/A	N/A
CirrHCV-176	Tissue	6 - Established Cirrhosis	no	N/A	N/A	N/A
CirrHCV-179	Tissue	6 - Established Cirrhosis	no	N/A	N/A	N/A
CirrHCV-200	Tissue	6 - Established Cirrhosis	no	N/A	N/A	N/A
CirrHCV-202	Tissue	6 - Established Cirrhosis	no	N/A	N/A	N/A
CirrHCV-216	Tissue	6 - Established Cirrhosis	no	N/A	N/A	N/A
CirrHCV-227	Tissue	6 - Established Cirrhosis	no	N/A	N/A	N/A
CirrHCV-238	Tissue	6 - Established Cirrhosis	no	N/A	N/A	N/A
CirrHCV-239	Tissue	6 - Established Cirrhosis	no	N/A	N/A	N/A
CirrHCV-243	Tissue	6 - Established Cirrhosis	no	N/A	N/A	N/A
CirrHCV-258N	Tissue	6 - Established Cirrhosis	no	N/A	N/A	N/A
CirrHCV-259N	Tissue	6 - Established Cirrhosis	no	N/A	N/A	N/A
CirrHCV-318N	Tissue	6 - Established Cirrhosis	no	N/A	N/A	N/A
CirrHCV-390N	Tissue	6 - Established Cirrhosis	no	N/A	N/A	N/A
CirrHCV-40N	Tissue	6 - Established Cirrhosis	no	N/A	N/A	N/A
CirrHCV-411N	Tissue	6 - Established Cirrhosis	no	N/A	N/A	N/A
CirrHCV-451	Tissue	6 - Established Cirrhosis	no	N/A	N/A	N/A
CirrHCV-452	Tissue	6 - Established Cirrhosis	no	N/A	N/A	N/A
CirrHCV-459	Tissue	6 - Established Cirrhosis	no	N/A	N/A	N/A
CirrHCV-465	Tissue	6 - Established Cirrhosis	no	N/A	N/A	N/A
CirrHCV-473	Tissue	6 - Established Cirrhosis	no	N/A	N/A	N/A
CirrHCV-516N	Tissue	6 - Established Cirrhosis	no	N/A	N/A	N/A
CirrHCV-525	Tissue	6 - Established Cirrhosis	no	N/A	N/A	N/A
CirrHCV-530	Tissue	6 - Established Cirrhosis	no	N/A	N/A	N/A
CirrHCV-56	Tissue	6 - Established Cirrhosis	no	N/A	N/A	N/A
CirrHCV-6	Tissue	6 - Established Cirrhosis	no	N/A	N/A	N/A
CirrHCV-617N	Tissue	6 - Established Cirrhosis	no	N/A	N/A	N/A
CirrHCV-621N	Tissue	6 - Established Cirrhosis	no	N/A	N/A	N/A
CirrHCV-661N	Tissue	6 - Established Cirrhosis	no	N/A	N/A	N/A
CirrHCV-673N	Tissue	6 - Established Cirrhosis	no	N/A	N/A	N/A
CirrHCV-693N	Tissue	6 - Established Cirrhosis	no	N/A	N/A	N/A
CirrHCV-7	Tissue	6 - Established Cirrhosis	no	N/A	N/A	N/A
Hbil-397T	Tissue	6 - Established Cirrhosis	yes	N/A	N/A	N/A
Hbil-552T	Tissue	6 - Established Cirrhosis	yes	N/A	N/A	N/A
HC-159	Tissue	6 - Established Cirrhosis	yes	N/A	N/A	N/A
HC-281T	Tissue	6 - Established Cirrhosis	yes	N/A	N/A	N/A
HC-419T	Tissue	6 - Established Cirrhosis	yes	N/A	N/A	N/A
HC-522T	Tissue	6 - Established Cirrhosis	yes	N/A	N/A	N/A
HC-527T	Tissue	6 - Established Cirrhosis	yes	N/A	N/A	N/A
HCCEtOH-110T	Tissue	6 - Established Cirrhosis	yes	N/A	N/A	N/A
HCCEtOH-157	Tissue	6 - Established Cirrhosis	yes	N/A	N/A	N/A
HCCEtOH-294T	Tissue	6 - Established Cirrhosis	yes	N/A	N/A	N/A
HCCEtOH-322T	Tissue	6 - Established Cirrhosis	yes	N/A	N/A	N/A
HCCEtOH-36	Tissue	6 - Established Cirrhosis	yes	N/A	N/A	N/A
HCCEtOH-415T	Tissue	6 - Established Cirrhosis	yes	N/A	N/A	N/A
HCCEtOH-444T	Tissue	6 - Established Cirrhosis	yes	N/A	N/A	N/A
HCCEtOH-466T	Tissue	6 - Established Cirrhosis	yes	N/A	N/A	N/A
HCCEtOH-515T	Tissue	6 - Established Cirrhosis	yes	N/A	N/A	N/A
HCCEtOH-562T	Tissue	6 - Established Cirrhosis	yes	N/A	N/A	N/A
HCCEtOH-576T	Tissue	6 - Established Cirrhosis	yes	N/A	N/A	N/A
HCCEtOH-59T	Tissue	6 - Established Cirrhosis	yes	N/A	N/A	N/A
HCCEtOH-608T	Tissue	6 - Established Cirrhosis	yes	N/A	N/A	N/A
HCCEtOH-649T	Tissue	6 - Established Cirrhosis	yes	N/A	N/A	N/A
HCCEtOH-665T	Tissue	6 - Established Cirrhosis	yes	N/A	N/A	N/A
HCCEtOH-674T	Tissue	6 - Established Cirrhosis	yes	N/A	N/A	N/A
HCCHCV-258T	Tissue	6 - Established Cirrhosis	yes	N/A	N/A	N/A
HCCHCV-259T	Tissue	6 - Established Cirrhosis	yes	N/A	N/A	N/A
HCCHCV-318T	Tissue	6 - Established Cirrhosis	yes	N/A	N/A	N/A
HCCHCV-390T	Tissue	6 - Established Cirrhosis	yes	N/A	N/A	N/A
HCCHCV-40T	Tissue	6 - Established Cirrhosis	yes	N/A	N/A	N/A
HCCHCV-411T	Tissue	6 - Established Cirrhosis	yes	N/A	N/A	N/A
HCCHCV-516T	Tissue	6 - Established Cirrhosis	yes	N/A	N/A	N/A
HCCHCV-617T	Tissue	6 - Established Cirrhosis	yes	N/A	N/A	N/A
HCCHCV-621T	Tissue	6 - Established Cirrhosis	yes	N/A	N/A	N/A
HCCHCV-661T	Tissue	6 - Established Cirrhosis	yes	N/A	N/A	N/A
HCCHCV-673T	Tissue	6 - Established Cirrhosis	yes	N/A	N/A	N/A
HCCHCV-693T	Tissue	6 - Established Cirrhosis	yes	N/A	N/A	N/A
HG-230T	Tissue	6 - Established Cirrhosis	yes	N/A	N/A	N/A
HG-563T	Tissue	6 - Established Cirrhosis	yes	N/A	N/A	N/A
HM-134T	Tissue	6 - Established Cirrhosis	yes	N/A	N/A	N/A
HM-212T	Tissue	6 - Established Cirrhosis	yes	N/A	N/A	N/A
HM-291T	Tissue	6 - Established Cirrhosis	yes	N/A	N/A	N/A
HM-300T	Tissue	6 - Established Cirrhosis	yes	N/A	N/A	N/A
HM-417T	Tissue	6 - Established Cirrhosis	yes	N/A	N/A	N/A
HM-520T	Tissue	6 - Established Cirrhosis	yes	N/A	N/A	N/A
HM-539T	Tissue	6 - Established Cirrhosis	yes	N/A	N/A	N/A
HM-540T	Tissue	6 - Established Cirrhosis	yes	N/A	N/A	N/A
TCGA-2V-A95S-01	Tissue	6 - Established Cirrhosis	yes	N/A	10793	N/A
TCGA-2Y-A9GS-01	Tissue	NA	yes	N/A	74	N/A
TCGA-2Y-A9GT-01	Tissue	NA	yes	N/A	17	N/A
TCGA-2Y-A9GU-01	Tissue	NA	yes	N/A	304	N/A
TCGA-2Y-A9GV-01	Tissue	NA	yes	N/A	6	N/A
TCGA-2Y-A9GW-01	Tissue	NA	yes	N/A	2	N/A
TCGA-2Y-A9GX-01	Tissue	NA	yes	N/A	1	N/A
TCGA-2Y-A9GY-01	Tissue	NA	yes	N/A	27600	N/A
TCGA-2Y-A9GZ-01	Tissue	NA	yes	N/A	7	N/A
TCGA-2Y-A9H0-01	Tissue	NA	yes	N/A	7598	N/A
TCGA-DD-AACW-01	Tissue	NA	yes	N/A	1726	N/A
TCGA-DD-AACX-01	Tissue	3,4 - Fibrous Speta	yes	N/A	3	N/A
TCGA-DD-AACY-01	Tissue	3,4 - Fibrous Speta	yes	N/A	290	N/A
TCGA-DD-AACZ-01	Tissue	NA	yes	N/A	25	N/A
TCGA-DD-AAD0-01	Tissue	NA	yes	N/A	4	N/A
TCGA-DD-AAD1-01	Tissue	NA	yes	N/A	451	N/A
TCGA-DD-AAD2-01	Tissue	1,2 - Portal Fibrosis	yes	N/A	25	N/A
TCGA-DD-AAD3-01	Tissue	1,2 - Portal Fibrosis	yes	N/A	NA	N/A
TCGA-DD-AAD5-01	Tissue	3,4 - Fibrous Speta	yes	N/A	73	N/A
TCGA-DD-AAD6-01	Tissue	NA	yes	N/A	NA	N/A
TCGA-DD-AAD8-01	Tissue	1,2 - Portal Fibrosis	yes	N/A	4	N/A
TCGA-DD-AADA-01	Tissue	NA	yes	N/A	8698	N/A
TCGA-DD-AADB-01	Tissue	NA	yes	N/A	419	N/A
TCGA-DD-AADC-01	Tissue	NA	yes	N/A	36	N/A
TCGA-DD-AADD-01	Tissue	NA	yes	N/A	9804	N/A
TCGA-DD-AADE-01	Tissue	3,4 - Fibrous Speta	yes	N/A	7	N/A
TCGA-DD-AADF-01	Tissue	NA	yes	N/A	725	N/A
TCGA-DD-AADG-01	Tissue	3,4 - Fibrous Speta	yes	N/A	2256	N/A
TCGA-DD-AADI-01	Tissue	NA	yes	N/A	27	N/A
TCGA-DD-AADJ-01	Tissue	NA	yes	N/A	708	N/A
TCGA-DD-AADK-01	Tissue	6 - Established Cirrhosis	yes	N/A	236	N/A
TCGA-DD-AADL-01	Tissue	NA	yes	N/A	14	N/A
TCGA-DD-AADM-01	Tissue	NA	yes	N/A	112	N/A
TCGA-DD-AADN-01	Tissue	NA	yes	N/A	151367	N/A
TCGA-DD-AADO-01	Tissue	NA	yes	N/A	176037	N/A
TCGA-DD-AADP-01	Tissue	NA	yes	N/A	9	N/A
TCGA-DD-AADQ-01	Tissue	NA	yes	N/A	7	N/A
TCGA-DD-AADR-01	Tissue	6 - Established Cirrhosis	yes	N/A	3	N/A
TCGA-DD-AADS-01	Tissue	NA	yes	N/A	11	N/A
TCGA-DD-AADU-01	Tissue	3,4 - Fibrous Speta	yes	N/A	52	N/A
TCGA-DD-AADV-01	Tissue	6 - Established Cirrhosis	yes	N/A	11	N/A
TCGA-DD-AADW-01	Tissue	6 - Established Cirrhosis	yes	N/A	250	N/A
TCGA-DD-AADY-01	Tissue	6 - Established Cirrhosis	yes	N/A	9	N/A
TCGA-DD-AAE0-01	Tissue	3,4 - Fibrous Speta	yes	N/A	8751	N/A
TCGA-DD-AAE1-01	Tissue	6 - Established Cirrhosis	yes	N/A	2693	N/A
TCGA-DD-AAE2-01	Tissue	6 - Established Cirrhosis	yes	N/A	41	N/A
TCGA-DD-AAE3-01	Tissue	1,2 - Portal Fibrosis	yes	N/A	2	N/A
TCGA-DD-AAE4-01	Tissue	6 - Established Cirrhosis	yes	N/A	7	N/A
TCGA-DD-AAE6-01	Tissue	6 - Established Cirrhosis	yes	N/A	113	N/A
TCGA-DD-AAE7-01	Tissue	3,4 - Fibrous Speta	yes	N/A	1	N/A
TCGA-DD-AAE8-01	Tissue	6 - Established Cirrhosis	yes	N/A	3	N/A
TCGA-DD-AAE9-01	Tissue	1,2 - Portal Fibrosis	yes	N/A	3	N/A
TCGA-DD-AAEA-01	Tissue	1,2 - Portal Fibrosis	yes	N/A	2	N/A
TCGA-DD-AAEB-01	Tissue	NA	yes	N/A	1	N/A
TCGA-DD-AAED-01	Tissue	1,2 - Portal Fibrosis	yes	N/A	2405	N/A
TCGA-DD-AAEE-01	Tissue	6 - Established Cirrhosis	yes	N/A	3	N/A
TCGA-DD-AAEG-01	Tissue	6 - Established Cirrhosis	yes	N/A	1	N/A
TCGA-DD-AAEH-01	Tissue	1,2 - Portal Fibrosis	yes	N/A	NA	N/A
TCGA-DD-AAEI-01	Tissue	6 - Established Cirrhosis	yes	N/A	24	N/A
TCGA-DD-AAEK-01	Tissue	6 - Established Cirrhosis	yes	N/A	5166	N/A
TCGA-DD-AAVP-01	Tissue	6 - Established Cirrhosis	yes	N/A	2	N/A
TCGA-DD-AAVQ-01	Tissue	6 - Established Cirrhosis	yes	N/A	1456	N/A
TCGA-DD-AAVR-01	Tissue	6 - Established Cirrhosis	yes	N/A	36	N/A
TCGA-DD-AAVS-01	Tissue	3,4 - Fibrous Speta	yes	N/A	50149	N/A
TCGA-DD-AAVU-01	Tissue	3,4 - Fibrous Speta	yes	N/A	2	N/A
TCGA-DD-AAVV-01	Tissue	3,4 - Fibrous Speta	yes	N/A	7	N/A
TCGA-DD-AAVW-01	Tissue	6 - Established Cirrhosis	yes	N/A	927	N/A
TCGA-DD-AAVX-01	Tissue	6 - Established Cirrhosis	yes	N/A	1	N/A
TCGA-DD-AAVY-01	Tissue	0 - No Fibrosis	yes	N/A	11	N/A
TCGA-DD-AAVZ-01	Tissue	6 - Established Cirrhosis	yes	N/A	945	N/A
TCGA-DD-AAW0-01	Tissue	3,4 - Fibrous Speta	yes	N/A	3	N/A
TCGA-DD-AAW1-01	Tissue	6 - Established Cirrhosis	yes	N/A	4	N/A
TCGA-DD-AAW2-01	Tissue	6 - Established Cirrhosis	yes	N/A	3	N/A
TCGA-DD-AAW3-01	Tissue	0 - No Fibrosis	yes	N/A	3	N/A
TCGA-ED-A459-01	Tissue	NA	yes	N/A	267	N/A
TCGA-ED-A4XI-01	Tissue	NA	yes	N/A	573	N/A
TCGA-ED-A5KG-01	Tissue	NA	yes	N/A	3177	N/A
TCGA-ED-A627-01	Tissue	NA	yes	N/A	NA	N/A
TCGA-ED-A66X-01	Tissue	NA	yes	N/A	5840	N/A
TCGA-ED-A66Y-01	Tissue	NA	yes	N/A	12100	N/A
TCGA-ED-A7PX-01	Tissue	NA	yes	N/A	24	N/A
TCGA-ED-A7PY-01	Tissue	NA	yes	N/A	2	N/A
TCGA-ED-A7PZ-01	Tissue	NA	yes	N/A	2	N/A
TCGA-ED-A7XO-01	Tissue	NA	yes	N/A	4	N/A
TCGA-ED-A7XP-01	Tissue	NA	yes	N/A	18	N/A
TCGA-ED-A82E-01	Tissue	NA	yes	N/A	4	N/A
TCGA-ED-A8O5-01	Tissue	NA	yes	N/A	498	N/A
TCGA-ED-A8O6-01	Tissue	NA	yes	N/A	3000	N/A
TCGA-ED-A97K-01	Tissue	NA	yes	N/A	3	N/A
TCGA-EP-A12J-01	Tissue	NA	yes	N/A	NA	N/A
TCGA-EP-A12J-11	Tissue	NA	no	N/A	NA	N/A
TCGA-EP-A26S-01	Tissue	0 - No Fibrosis	yes	N/A	NA	N/A
TCGA-EP-A26S-11	Tissue	0 - No Fibrosis	no	N/A	NA	N/A
TCGA-EP-A2KA-01	Tissue	1,2 - Portal Fibrosis	yes	N/A	13	N/A
TCGA-EP-A2KB-01	Tissue	NA	yes	N/A	2128	N/A
TCGA-EP-A2KC-01	Tissue	NA	yes	N/A	43	N/A
TCGA-EP-A3JL-01	Tissue	NA	yes	N/A	13	N/A
TCGA-EP-A3RK-01	Tissue	1,2 - Portal Fibrosis	yes	N/A	7	N/A
TCGA-ES-A2HS-01	Tissue	0 - No Fibrosis	yes	N/A	1	N/A

TCGA-2Y-A9H1-01	Tissue	1,2 - Portal Fibrosis	yes	N/A	3	N/A
TCGA-2Y-A9H2-01	Tissue	NA	yes	N/A	2	N/A
TCGA-2Y-A9H3-01	Tissue	1,2 - Portal Fibrosis	yes	N/A	5640	N/A
TCGA-2Y-A9H4-01	Tissue	NA	yes	N/A	11	N/A
TCGA-2Y-A9H5-01	Tissue	1,2 - Portal Fibrosis	yes	N/A	11700	N/A
TCGA-2Y-A9H6-01	Tissue	NA	yes	N/A	114	N/A
TCGA-2Y-A9H7-01	Tissue	3,4 - Fibrous Speta	yes	N/A	6	N/A
TCGA-2Y-A9H8-01	Tissue	NA	yes	N/A	234000	N/A
TCGA-2Y-A9H9-01	Tissue	NA	yes	N/A	12	N/A
TCGA-2Y-A9HA-01	Tissue	3,4 - Fibrous Speta	yes	N/A	114	N/A
TCGA-2Y-A9HB-01	Tissue	NA	yes	N/A	21	N/A
TCGA-3K-AAZ8-01	Tissue	5 - Nodular Formation and Incomplete Cirrhosis	yes	N/A	NA	N/A
TCGA-4R-AA8I-01	Tissue	6 - Established Cirrhosis	yes	N/A	5	N/A
TCGA-5C-A9VG-01	Tissue	NA	yes	N/A	NA	N/A
TCGA-5C-A9VH-01	Tissue	NA	yes	N/A	NA	N/A
TCGA-5C-AAPD-01	Tissue	NA	yes	N/A	NA	N/A
TCGA-5R-AA1C-01	Tissue	1,2 - Portal Fibrosis	yes	N/A	2	N/A
TCGA-5R-AA1D-01	Tissue	0 - No Fibrosis	yes	N/A	5	N/A
TCGA-5R-AAAM-01	Tissue	6 - Established Cirrhosis	yes	N/A	32	N/A
TCGA-BC-4072-01	Tissue	NA	yes	N/A	NA	N/A
TCGA-BC-4073-01	Tissue	NA	yes	N/A	NA	N/A
TCGA-BC-A10Q-01	Tissue	NA	yes	N/A	NA	N/A
TCGA-BC-A10Q-11	Tissue	NA	no	N/A	NA	N/A
TCGA-BC-A10R-01	Tissue	NA	yes	N/A	27	N/A
TCGA-BC-A10R-11	Tissue	NA	no	N/A	27	N/A
TCGA-BC-A10S-01	Tissue	NA	yes	N/A	NA	N/A
TCGA-BC-A10S-11	Tissue	NA	no	N/A	NA	N/A
TCGA-BC-A10T-01	Tissue	1,2 - Portal Fibrosis	yes	N/A	NA	N/A
TCGA-BC-A10T-11	Tissue	1,2 - Portal Fibrosis	no	N/A	NA	N/A
TCGA-BC-A10U-01	Tissue	NA	yes	N/A	38	N/A
TCGA-BC-A10U-11	Tissue	NA	no	N/A	38	N/A
TCGA-BC-A10W-01	Tissue	NA	yes	N/A	79	N/A
TCGA-BC-A10W-11	Tissue	NA	no	N/A	79	N/A
TCGA-BC-A10X-01	Tissue	NA	yes	N/A	264	N/A
TCGA-BC-A10X-11	Tissue	NA	no	N/A	264	N/A
TCGA-BC-A10Y-01	Tissue	NA	yes	N/A	10575	N/A
TCGA-BC-A10Y-11	Tissue	NA	no	N/A	10575	N/A
TCGA-BC-A10Z-01	Tissue	NA	yes	N/A	779	N/A
TCGA-BC-A10Z-11	Tissue	NA	no	N/A	779	N/A
TCGA-BC-A110-01	Tissue	NA	yes	N/A	NA	N/A
TCGA-BC-A110-11	Tissue	NA	no	N/A	NA	N/A
TCGA-BC-A112-01	Tissue	0 - No Fibrosis	yes	N/A	233	N/A
TCGA-BC-A112-11	Tissue	0 - No Fibrosis	no	N/A	233	N/A
TCGA-BC-A216-01	Tissue	0 - No Fibrosis	yes	N/A	831	N/A
TCGA-BC-A216-11	Tissue	0 - No Fibrosis	no	N/A	831	N/A
TCGA-BC-A217-01	Tissue	0 - No Fibrosis	yes	N/A	28	N/A
TCGA-BC-A3KF-01	Tissue	NA	yes	N/A	NA	N/A
TCGA-BC-A3KG-01	Tissue	NA	yes	N/A	618	N/A
TCGA-BC-A5W4-01	Tissue	NA	yes	N/A	NA	N/A
TCGA-BC-A69H-01	Tissue	NA	yes	N/A	103900	N/A
TCGA-BC-A69I-01	Tissue	NA	yes	N/A	2	N/A
TCGA-BC-A8YO-01	Tissue	NA	yes	N/A	NA	N/A
TCGA-BD-A2L6-01	Tissue	3,4 - Fibrous Speta	yes	N/A	53	N/A
TCGA-BD-A2L6-11	Tissue	3,4 - Fibrous Speta	no	N/A	53	N/A
TCGA-BD-A3EP-01	Tissue	1,2 - Portal Fibrosis	yes	N/A	17	N/A
TCGA-BD-A3EP-11	Tissue	1,2 - Portal Fibrosis	no	N/A	17	N/A
TCGA-BD-A3ER-01	Tissue	6 - Established Cirrhosis	yes	N/A	5	N/A
TCGA-BW-A5NO-01	Tissue	3,4 - Fibrous Speta	yes	N/A	NA	N/A
TCGA-BW-A5NP-01	Tissue	3,4 - Fibrous Speta	yes	N/A	143684	N/A
TCGA-BW-A5NQ-01	Tissue	6 - Established Cirrhosis	yes	N/A	NA	N/A
TCGA-CC-5258-01	Tissue	NA	yes	N/A	NA	N/A
TCGA-CC-5259-01	Tissue	NA	yes	N/A	NA	N/A
TCGA-CC-5260-01	Tissue	NA	yes	N/A	NA	N/A
TCGA-CC-5261-01	Tissue	NA	yes	N/A	NA	N/A
TCGA-CC-5262-01	Tissue	NA	yes	N/A	NA	N/A
TCGA-CC-5263-01	Tissue	NA	yes	N/A	NA	N/A
TCGA-CC-5264-01	Tissue	NA	yes	N/A	NA	N/A
TCGA-CC-A123-01	Tissue	NA	yes	N/A	NA	N/A
TCGA-CC-A1HT-01	Tissue	NA	yes	N/A	NA	N/A
TCGA-CC-A3M9-01	Tissue	NA	yes	N/A	NA	N/A
TCGA-CC-A3MA-01	Tissue	NA	yes	N/A	NA	N/A
TCGA-CC-A3MB-01	Tissue	NA	yes	N/A	NA	N/A
TCGA-CC-A3MC-01	Tissue	NA	yes	N/A	NA	N/A
TCGA-CC-A5UC-01	Tissue	NA	yes	N/A	NA	N/A
TCGA-CC-A5UD-01	Tissue	NA	yes	N/A	NA	N/A
TCGA-CC-A5UE-01	Tissue	NA	yes	N/A	NA	N/A
TCGA-CC-A7IE-01	Tissue	NA	yes	N/A	NA	N/A
TCGA-CC-A7IF-01	Tissue	NA	yes	N/A	NA	N/A
TCGA-CC-A7IG-01	Tissue	NA	yes	N/A	NA	N/A
TCGA-CC-A7IH-01	Tissue	NA	yes	N/A	NA	N/A
TCGA-CC-A7II-01	Tissue	NA	yes	N/A	NA	N/A
TCGA-CC-A7IJ-01	Tissue	NA	yes	N/A	NA	N/A
TCGA-CC-A7IK-01	Tissue	NA	yes	N/A	NA	N/A
TCGA-CC-A7IL-01	Tissue	NA	yes	N/A	NA	N/A
TCGA-CC-A8HS-01	Tissue	NA	yes	N/A	NA	N/A
TCGA-CC-A8HT-01	Tissue	NA	yes	N/A	NA	N/A
TCGA-CC-A8HU-01	Tissue	NA	yes	N/A	NA	N/A
TCGA-CC-A8HV-01	Tissue	NA	yes	N/A	NA	N/A
TCGA-CC-A9FS-01	Tissue	NA	yes	N/A	NA	N/A
TCGA-ES-A2HS-11	Tissue	0 - No Fibrosis	no	N/A	1	N/A
TCGA-ES-A2HT-01	Tissue	0 - No Fibrosis	yes	N/A	4	N/A
TCGA-ES-A2HT-11	Tissue	0 - No Fibrosis	no	N/A	4	N/A
TCGA-FV-A23B-01	Tissue	NA	yes	N/A	NA	N/A
TCGA-FV-A23B-11	Tissue	NA	no	N/A	NA	N/A
TCGA-FV-A2QQ-01	Tissue	NA	yes	N/A	NA	N/A
TCGA-FV-A2QR-01	Tissue	NA	yes	N/A	NA	N/A
TCGA-FV-A2QR-11	Tissue	NA	no	N/A	NA	N/A
TCGA-FV-A3I0-01	Tissue	NA	yes	N/A	NA	N/A
TCGA-FV-A3I1-01	Tissue	0 - No Fibrosis	yes	N/A	NA	N/A
TCGA-FV-A3R2-01	Tissue	NA	yes	N/A	NA	N/A
TCGA-FV-A3R3-01	Tissue	NA	yes	N/A	NA	N/A
TCGA-FV-A495-01	Tissue	3,4 - Fibrous Speta	yes	N/A	39	N/A
TCGA-FV-A496-01	Tissue	0 - No Fibrosis	yes	N/A	67	N/A
TCGA-FV-A4ZP-01	Tissue	NA	yes	N/A	20326	N/A
TCGA-FV-A4ZQ-01	Tissue	NA	yes	N/A	NA	N/A
TCGA-G3-A25S-01	Tissue	6 - Established Cirrhosis	yes	N/A	281	N/A
TCGA-G3-A25T-01	Tissue	0 - No Fibrosis	yes	N/A	7	N/A
TCGA-G3-A25U-01	Tissue	0 - No Fibrosis	yes	N/A	10	N/A
TCGA-G3-A25V-01	Tissue	6 - Established Cirrhosis	yes	N/A	15	N/A
TCGA-G3-A25W-01	Tissue	0 - No Fibrosis	yes	N/A	6	N/A
TCGA-G3-A25W-11	Tissue	0 - No Fibrosis	no	N/A	6	N/A
TCGA-G3-A25X-01	Tissue	1,2 - Portal Fibrosis	yes	N/A	11	N/A
TCGA-G3-A25X-11	Tissue	1,2 - Portal Fibrosis	no	N/A	11	N/A
TCGA-G3-A25Y-01	Tissue	3,4 - Fibrous Speta	yes	N/A	35	N/A
TCGA-DD-A1EC-01	Tissue	0 - No Fibrosis	yes	N/A	2	N/A
TCGA-G3-A3CG-01	Tissue	6 - Established Cirrhosis	yes	N/A	67	N/A
TCGA-G3-A3CH-01	Tissue	6 - Established Cirrhosis	yes	N/A	41	N/A
TCGA-G3-A3CI-01	Tissue	0 - No Fibrosis	yes	N/A	2	N/A
TCGA-DD-A1EC-11	Tissue	0 - No Fibrosis	no	N/A	2	N/A
TCGA-G3-A3CK-01	Tissue	6 - Established Cirrhosis	yes	N/A	3	N/A
TCGA-G3-A5SI-01	Tissue	6 - Established Cirrhosis	yes	N/A	9	N/A
TCGA-G3-A5SJ-01	Tissue	6 - Established Cirrhosis	yes	N/A	3	N/A
TCGA-G3-A5SK-01	Tissue	6 - Established Cirrhosis	yes	N/A	NA	N/A
TCGA-G3-A5SL-01	Tissue	0 - No Fibrosis	yes	N/A	4	N/A
TCGA-G3-A5SM-01	Tissue	6 - Established Cirrhosis	yes	N/A	12	N/A
TCGA-G3-A6UC-01	Tissue	6 - Established Cirrhosis	yes	N/A	4	N/A
TCGA-G3-A7M5-01	Tissue	0 - No Fibrosis	yes	N/A	6	N/A
TCGA-G3-A7M6-01	Tissue	1,2 - Portal Fibrosis	yes	N/A	24	N/A
TCGA-G3-A7M7-01	Tissue	3,4 - Fibrous Speta	yes	N/A	4	N/A
TCGA-G3-A7M8-01	Tissue	6 - Established Cirrhosis	yes	N/A	5	N/A
TCGA-G3-A7M9-01	Tissue	6 - Established Cirrhosis	yes	N/A	22868	N/A
TCGA-G3-AAUZ-01	Tissue	1,2 - Portal Fibrosis	yes	N/A	3	N/A
TCGA-G3-AAV0-01	Tissue	0 - No Fibrosis	yes	N/A	4	N/A
TCGA-G3-AAV1-01	Tissue	6 - Established Cirrhosis	yes	N/A	8	N/A
TCGA-G3-AAV2-01	Tissue	6 - Established Cirrhosis	yes	N/A	4	N/A
TCGA-G3-AAV3-01	Tissue	6 - Established Cirrhosis	yes	N/A	5	N/A
TCGA-G3-AAV4-01	Tissue	3,4 - Fibrous Speta	yes	N/A	4	N/A
TCGA-G3-AAV5-01	Tissue	6 - Established Cirrhosis	yes	N/A	NA	N/A
TCGA-G3-AAV6-01	Tissue	0 - No Fibrosis	yes	N/A	57875	N/A
TCGA-DD-A1ED-01	Tissue	0 - No Fibrosis	yes	N/A	3	N/A
TCGA-GJ-A3OU-01	Tissue	NA	yes	N/A	8	N/A
TCGA-GJ-A6C0-01	Tissue	NA	yes	N/A	NA	N/A
TCGA-GJ-A9DB-01	Tissue	NA	yes	N/A	NA	N/A
TCGA-HP-A5MZ-01	Tissue	NA	yes	N/A	NA	N/A
TCGA-HP-A5N0-01	Tissue	0 - No Fibrosis	yes	N/A	NA	N/A
TCGA-K7-A5RF-01	Tissue	NA	yes	N/A	5	N/A
TCGA-K7-A5RG-01	Tissue	NA	yes	N/A	19	N/A
TCGA-K7-A6G5-01	Tissue	NA	yes	N/A	NA	N/A
TCGA-K7-AAU7-01	Tissue	NA	yes	N/A	290	N/A
TCGA-KR-A7K0-01	Tissue	NA	yes	N/A	11	N/A
TCGA-KR-A7K2-01	Tissue	NA	yes	N/A	14	N/A
TCGA-KR-A7K7-01	Tissue	NA	yes	N/A	308836	N/A
TCGA-KR-A7K8-01	Tissue	NA	yes	N/A	154	N/A
TCGA-LG-A6GG-01	Tissue	NA	yes	N/A	NA	N/A
TCGA-LG-A9QC-01	Tissue	NA	yes	N/A	NA	N/A
TCGA-LG-A9QD-01	Tissue	NA	yes	N/A	NA	N/A
TCGA-MI-A75C-01	Tissue	NA	yes	N/A	4	N/A
TCGA-MI-A75E-01	Tissue	1,2 - Portal Fibrosis	yes	N/A	5	N/A
TCGA-MI-A75G-01	Tissue	6 - Established Cirrhosis	yes	N/A	6	N/A
TCGA-MI-A75H-01	Tissue	6 - Established Cirrhosis	yes	N/A	11	N/A
TCGA-MI-A75I-01	Tissue	NA	yes	N/A	NA	N/A
TCGA-MR-A520-01	Tissue	NA	yes	N/A	NA	N/A
TCGA-MR-A8JO-01	Tissue	NA	yes	N/A	2	N/A
TCGA-NI-A4U2-01	Tissue	1,2 - Portal Fibrosis	yes	N/A	5	N/A
TCGA-NI-A8LF-01	Tissue	NA	yes	N/A	NA	N/A
TCGA-O8-A75V-01	Tissue	6 - Established Cirrhosis	yes	N/A	7	N/A
TCGA-PD-A5DF-01	Tissue	NA	yes	N/A	NA	N/A
TCGA-QA-A7B7-01	Tissue	0 - No Fibrosis	yes	N/A	1751	N/A
TCGA-RC-A6M3-01	Tissue	NA	yes	N/A	NA	N/A
TCGA-RC-A6M4-01	Tissue	NA	yes	N/A	5	N/A
TCGA-RC-A6M5-01	Tissue	NA	yes	N/A	NA	N/A
TCGA-RC-A6M6-01	Tissue	NA	yes	N/A	12986	N/A
TCGA-RC-A7S9-01	Tissue	6 - Established Cirrhosis	yes	N/A	10	N/A
TCGA-RC-A7SB-01	Tissue	0 - No Fibrosis	yes	N/A	3	N/A
TCGA-RC-A7SF-01	Tissue	3,4 - Fibrous Speta	yes	N/A	87	N/A
TCGA-RC-A7SH-01	Tissue	3,4 - Fibrous Speta	yes	N/A	2466	N/A
TCGA-RC-A7SK-01	Tissue	1,2 - Portal Fibrosis	yes	N/A	25	N/A
TCGA-RG-A7D4-01	Tissue	NA	yes	N/A	5650	N/A

TCGA-CC-A9FU-01	Tissue	NA	yes	N/A	NA	N/A
TCGA-CC-A9FV-01	Tissue	NA	yes	N/A	NA	N/A
TCGA-CC-A9FW-01	Tissue	NA	yes	N/A	NA	N/A
TCGA-DD-A113-01	Tissue	0 - No Fibrosis	yes	N/A	133	N/A
TCGA-DD-A113-11	Tissue	0 - No Fibrosis	no	N/A	133	N/A
TCGA-DD-A114-01	Tissue	5 - Nodular Formation and Incomplete Cirrhosis	yes	N/A	21	N/A
TCGA-DD-A114-11	Tissue	5 - Nodular Formation and Incomplete Cirrhosis	no	N/A	21	N/A
TCGA-DD-A115-01	Tissue	0 - No Fibrosis	yes	N/A	10	N/A
TCGA-DD-A115-11	Tissue	0 - No Fibrosis	no	N/A	10	N/A
TCGA-DD-A116-01	Tissue	3,4 - Fibrous Speta	yes	N/A	24	N/A
TCGA-DD-A116-11	Tissue	3,4 - Fibrous Speta	no	N/A	24	N/A
TCGA-DD-A118-01	Tissue	0 - No Fibrosis	yes	N/A	85150	N/A
TCGA-DD-A118-11	Tissue	0 - No Fibrosis	no	N/A	85150	N/A
TCGA-DD-A119-01	Tissue	0 - No Fibrosis	yes	N/A	1836	N/A
TCGA-DD-A119-11	Tissue	0 - No Fibrosis	no	N/A	1836	N/A
TCGA-DD-A11A-01	Tissue	0 - No Fibrosis	yes	N/A	223	N/A
TCGA-DD-A11A-11	Tissue	0 - No Fibrosis	no	N/A	223	N/A
TCGA-DD-A11B-01	Tissue	6 - Established Cirrhosis	yes	N/A	15	N/A
TCGA-DD-A11B-11	Tissue	6 - Established Cirrhosis	no	N/A	15	N/A
TCGA-DD-A11C-01	Tissue	0 - No Fibrosis	yes	N/A	11	N/A
TCGA-DD-A11C-11	Tissue	0 - No Fibrosis	no	N/A	11	N/A
TCGA-DD-A11D-01	Tissue	6 - Established Cirrhosis	yes	N/A	8	N/A
TCGA-DD-A11D-11	Tissue	6 - Established Cirrhosis	no	N/A	8	N/A
TCGA-DD-A1E9-01	Tissue	6 - Established Cirrhosis	yes	N/A	202	N/A
TCGA-DD-A1E9-11	Tissue	6 - Established Cirrhosis	no	N/A	202	N/A
TCGA-DD-A1EA-01	Tissue	1,2 - Portal Fibrosis	yes	N/A	3	N/A
TCGA-ZP-A9D1-01	Tissue	5 - Nodular Formation and Incomplete Cirrhosis	yes	N/A	8	N/A
TCGA-ZS-A9CD-01	Tissue	5 - Nodular Formation and Incomplete Cirrhosis	yes	N/A	NA	N/A
TCGA-G3-A25Z-01	Tissue	5 - Nodular Formation and Incomplete Cirrhosis	yes	N/A	44	N/A
TCGA-G3-A3CJ-01	Tissue	5 - Nodular Formation and Incomplete Cirrhosis	yes	N/A	3	N/A
TCGA-G3-AAV7-01	Tissue	5 - Nodular Formation and Incomplete Cirrhosis	yes	N/A	3	N/A
TCGA-DD-A4NA-01	Tissue	5 - Nodular Formation and Incomplete Cirrhosis	yes	N/A	3	N/A
TCGA-DD-AACI-01	Tissue	5 - Nodular Formation and Incomplete Cirrhosis	yes	N/A	1	N/A
TCGA-T1-A6J8-01	Tissue	NA	yes	N/A	NA	N/A
TCGA-UB-A7MA-01	Tissue	0 - No Fibrosis	yes	N/A	2008	N/A
TCGA-UB-A7MB-01	Tissue	0 - No Fibrosis	yes	N/A	1865	N/A
TCGA-UB-A7MC-01	Tissue	1,2 - Portal Fibrosis	yes	N/A	126	N/A
TCGA-UB-A7MD-01	Tissue	6 - Established Cirrhosis	yes	N/A	23	N/A
TCGA-UB-A7ME-01	Tissue	1,2 - Portal Fibrosis	yes	N/A	1388	N/A
TCGA-UB-A7MF-01	Tissue	6 - Established Cirrhosis	yes	N/A	2505	N/A
TCGA-UB-AA0U-01	Tissue	0 - No Fibrosis	yes	N/A	26	N/A
TCGA-UB-AA0V-01	Tissue	0 - No Fibrosis	yes	N/A	3	N/A
TCGA-WJ-A86L-01	Tissue	0 - No Fibrosis	yes	N/A	16	N/A
TCGA-WQ-A9G7-01	Tissue	NA	yes	N/A	NA	N/A
TCGA-WQ-AB4B-01	Tissue	NA	yes	N/A	5	N/A
TCGA-WX-AA44-01	Tissue	6 - Established Cirrhosis	yes	N/A	48	N/A
TCGA-WX-AA46-01	Tissue	0 - No Fibrosis	yes	N/A	1	N/A
TCGA-WX-AA47-01	Tissue	0 - No Fibrosis	yes	N/A	1	N/A
TCGA-XR-A8TC-01	Tissue	6 - Established Cirrhosis	yes	N/A	47	N/A
TCGA-XR-A8TD-01	Tissue	0 - No Fibrosis	yes	N/A	188	N/A
TCGA-XR-A8TE-01	Tissue	0 - No Fibrosis	yes	N/A	809	N/A
TCGA-XR-A8TF-01	Tissue	3,4 - Fibrous Speta	yes	N/A	39	N/A
TCGA-XR-A8TG-01	Tissue	6 - Established Cirrhosis	yes	N/A	1	N/A
TCGA-YA-A8S7-01	Tissue	NA	yes	N/A	56	N/A
TCGA-ZP-A9CV-01	Tissue	NA	yes	N/A	48	N/A
TCGA-ZP-A9CY-01	Tissue	6 - Established Cirrhosis	yes	N/A	NA	N/A
TCGA-ZP-A9CZ-01	Tissue	3,4 - Fibrous Speta	yes	N/A	8	N/A
TCGA-ZP-A9D0-01	Tissue	0 - No Fibrosis	yes	N/A	NA	N/A
TCGA-DD-A1ED-11	Tissue	0 - No Fibrosis	no	N/A	3	N/A
TCGA-ZP-A9D2-01	Tissue	1,2 - Portal Fibrosis	yes	N/A	NA	N/A
TCGA-ZP-A9D4-01	Tissue	0 - No Fibrosis	yes	N/A	2	N/A
TCGA-DD-A1EE-01	Tissue	6 - Established Cirrhosis	yes	N/A	3	N/A
TCGA-ZS-A9CE-01	Tissue	0 - No Fibrosis	yes	N/A	NA	N/A
TCGA-ZS-A9CF-01	Tissue	0 - No Fibrosis	yes	N/A	NA	N/A
TCGA-ZS-A9CF-02	Tissue	0 - No Fibrosis	yes	N/A	NA	N/A
TCGA-ZS-A9CG-01	Tissue	0 - No Fibrosis	yes	N/A	NA	N/A

Table S2 : Clinical information for cfDNA samples used in bisulfite pyrosequencing.

Sample_ID	Cirrhosis	HCC	MELD	AFP
20535	Yes	No	18	3
20563	Yes	No	30	1.9
20600	Yes	No	8	3.4
20646	Yes	No	14	5.7
20736	Yes	No	11	5.1
20739	Yes	No	10	1.2
20775	Yes	No	6	6.5
20864	Yes	No	11	7.6
20918	Yes	No	15	2
20987	Yes	No	17	2.5
20361	Yes	Yes	11	102
20642	Yes	Yes	7	3.1
19910	Yes	Yes	10	36
19657	Yes	Yes	8	6
19249	Yes	Yes	9	6.2
18298	Yes	Yes	12	82258
19784	Yes	Yes	11	186
19888	Yes	Yes	17	2.6
20738	Yes	Yes	23	3
21440	Yes	Yes	10	3.5
HBC-011	yes	yes	7	2.1
HBC-010	yes	yes	6	8.5
HBC-013	yes	yes	10	11.7
HBC-021	yes	yes	8	5.6
HBC-008	yes	yes	28	4.4
HBC-086	yes	yes	8	18.8
HBC-012	yes	yes	10	1019.1
HBC-018	yes	yes	28	2.5
HBC-036	yes	yes	14	2.9
HBC-007	yes	yes	10	7.7
HBC-1338	yes	no	19	2.7
HBC-1062	yes	no	11	4.4
HBC-1005	yes	no	14	2.2
HBC-1073	yes	no	11	5
HBC-1023	yes	no	9	2.4
HBC-1342	yes	no	10	2.3
HBC-1334	yes	no	11	4.4
HBC-1115	yes	no	11	5.3
HBC-1054	yes	no	10	3.3
HBC-1036	yes	no	9	5.9

Table S3: Primers used in this study for bisulfite pyrosequencing.

cg07610777-F	TTGGGGTAAGAAAATAGTTGTATGG
cg07610777-Rbio	/5biosg/ACCTCAATATCCCCTAAATAAA
cg07610777-seq	TATAAAGTTTATTTGAGGGAAAAGAA
cg13781744-F	AAGAGTTAGGGTGGGAGTGAGAG
cg13781744-Rbio	/5biosg/CACCTAAAAACTCACCCTAAAAACA
cg13781744-seq	TTAGGGTTGGTTTTTTTTAT
cg04645914-F	AGTGATTGAGGTGTTTTGAA
cg04645914-Rbio	AAAAACCCGAAAAAACTATTACCAC
cg04645914-seq	GGTGTTTTGAAATTAATTTATTGTT
cg18054281-F	GAGTTGTAAAGGATTTATGTTTTTT
cg18054281-Rbio	/5biosg/CATATAAACCTATATACCAAATCTCC
cg18054281-seq	AAGTATTTGTAAGAGTTTTAAG
cg25026480-F	GGGTTTTTTCGGTTATAGTTTTTTT
cg25026480-Rbio	/5biosg/TAACAACCCGAATAAATCACAAACT
cg25026480-seq	GGGTTTGTGTTGTTTTTTTG

Table S4: Hypermethylated CpGs used for Lasso regression analysis.

cg00145955	cg03304019	cg06192883	cg08805054	cg13673023	cg16899036	cg20071427	cg24437859
cg00233028	cg03354717	cg06202802	cg09050331	cg13735469	cg17143376	cg20073313	cg24528164
cg00255919	cg03368099	cg06204126	cg09082487	cg13781744	cg17153082	cg20337103	cg24564492
cg00298977	cg03435448	cg06215569	cg09183450	cg13818451	cg17179862	cg20356637	cg24642468
cg00337187	cg03455424	cg06223172	cg09234936	cg13900817	cg17200137	cg20426959	cg24696332
cg00347757	cg03473127	cg06343169	cg09248054	cg13954457	cg17319294	cg20570109	cg24711774
cg00376654	cg03589820	cg06351481	cg09259772	cg14042308	cg17417856	cg20600729	cg24718722
cg00392156	cg03651292	cg06416034	cg09308553	cg14058329	cg17450621	cg20985399	cg24768135
cg00414835	cg03706056	cg06478886	cg09327855	cg14066993	cg17453456	cg21144063	cg24818238
cg00423675	cg03739573	cg06488443	cg09827751	cg14093255	cg17478893	cg21209395	cg24834889
cg00524708	cg03803541	cg06496484	cg09862733	cg14095959	cg17569124	cg21209859	cg25019989
cg00607058	cg03803861	cg06578342	cg09916840	cg14114910	cg17704343	cg21287054	cg25035485
cg00782355	cg03917138	cg06638795	cg09973676	cg14361804	cg17713488	cg21334510	cg25299214
cg00795812	cg03922251	cg06687640	cg10052687	cg14421582	cg17735531	cg21366673	cg25468005
cg00836482	cg03928874	cg06834661	cg10143146	cg14442890	cg17822325	cg21415530	cg25468618
cg00926420	cg04003990	cg06853416	cg10258719	cg14459158	cg17847520	cg21540359	cg25599129
cg00980581	cg04024827	cg06874426	cg10287485	cg14541011	cg18014109	cg21548955	cg25618731
cg00983520	cg04090468	cg06974930	cg10380221	cg14601038	cg18032014	cg21559386	cg25651476
cg00986350	cg04117972	cg07026448	cg10553748	cg14612417	cg18059012	cg21577836	cg25924688
cg01076129	cg04175111	cg07028396	cg10709021	cg14736365	cg18305365	cg21751145	cg26035892
cg01138706	cg04193820	cg07028768	cg10722410	cg14740554	cg18358723	cg21785034	cg26152754
cg01211283	cg04255230	cg07100700	cg10750464	cg14899046	cg18428234	cg21811284	cg26224785
cg01223400	cg04359418	cg07192048	cg10841673	cg14988722	cg18437808	cg21877680	cg26385126
cg01278041	cg04459086	cg07248377	cg11003309	cg15006627	cg18442187	cg21885361	cg26445985
cg01290755	cg04509559	cg07292311	cg11003725	cg15044767	cg18464402	cg21908235	cg26514578
cg01403532	cg04574034	cg07388493	cg11045746	cg15054274	cg18477429	cg21920469	cg26526883
cg01489256	cg04610274	cg07389611	cg11113753	cg15243034	cg18486590	cg21937660	cg26597838
cg01578057	cg04612030	cg07390210	cg11168433	cg15498409	cg18489675	cg22126965	cg26606589
cg01773927	cg04645914	cg07402062	cg11205312	cg15505700	cg18512515	cg22151446	cg26609120
cg01802772	cg04687439	cg07407736	cg11320910	cg15555527	cg18628367	cg22175873	cg26643967
cg01810575	cg04755771	cg07471943	cg11418303	cg15734230	cg18850089	cg22532774	cg26673980
cg01946191	cg04767897	cg07479615	cg11521079	cg15776494	cg18864882	cg22542803	cg26676440
cg01971227	cg04771946	cg07485775	cg11631063	cg15816012	cg18919684	cg22632756	cg26697334
cg02121547	cg04781080	cg07519485	cg11668053	cg15844419	cg18920097	cg22632947	cg26701815
cg02192673	cg04857710	cg07584066	cg11714502	cg16003790	cg19021236	cg22702351	cg26734888
cg02230254	cg04876069	cg07610777	cg11730254	cg16009311	cg19021985	cg22917372	cg26740937
cg02261771	cg04889069	cg07612655	cg11837181	cg16079430	cg19135362	cg22928954	cg26750617
cg02264895	cg04902871	cg07629625	cg11935831	cg16101008	cg19158754	cg23049448	cg26823505
cg02284273	cg04917181	cg07669403	cg12186618	cg16118803	cg19223119	cg23287992	cg26846409
cg02289752	cg04979435	cg07674570	cg12495090	cg16120147	cg19389372	cg23319460	cg26863172
cg02457346	cg04992150	cg07683388	cg12534390	cg16121206	cg19401111	cg23429510	cg26917745
cg02464608	cg04998327	cg07812957	cg12537405	cg16148134	cg19432993	cg23521973	cg27019019
cg02499614	cg05031011	cg07875786	cg12615766	cg16170346	cg19433091	cg23548627	cg27024272
cg02580250	cg05135176	cg07915516	cg12672189	cg16180796	cg19470812	cg23574719	cg27127887
cg02594677	cg05325887	cg07967228	cg12732514	cg16231917	cg19536929	cg23634025	cg27169020
cg02597246	cg05399718	cg08000731	cg12759554	cg16310958	cg19666541	cg23648082	cg27364244
cg02638589	cg05506011	cg08010094	cg12894984	cg16312900	cg19689322	cg23663760	cg27408897
cg02852398	cg05548190	cg08351464	cg13105599	cg16327477	cg19699056	cg23740758	cg27464846
cg02888166	cg05730108	cg08355659	cg13127920	cg16435923	cg19728382	cg23758822	cg27534828
cg02962952	cg05783384	cg08415582	cg13206220	cg16541275	cg19771372	cg23896972	cg27583010
cg03020684	cg05810848	cg08496033	cg13247663	cg16620608	cg19800670	cg23974730	cg27596890
cg03044367	cg05820861	cg08504662	cg13308137	cg16649560	cg19856897	cg24035043	
cg03110082	cg05938149	cg08543278	cg13330868	cg16664193	cg19866811	cg24040502	
cg03188948	cg06002687	cg08717807	cg13370460	cg16725094	cg19970908	cg24266485	
cg03206537	cg06015422	cg08719486	cg13492364	cg16792363	cg20043937	cg24310959	
cg03269716	cg06106242	cg08734931	cg13574337	cg16817237	cg20048521	cg24433189	

Table S5: Hypomethylated CpGs used for Lasso regression analysis.

cg18537454	cg23447239	cg11347165	cg12126038	cg23504522	cg01498832	cg03834411
cg19946376	cg04713352	cg15580458	cg27372170	cg10954330	cg13335329	cg03940883
cg10444224	cg18054281	cg08571738	cg09300795	cg02920045	cg22337626	cg25977769
cg25026480	cg07769015	cg11418607	cg18669823	cg15124926	cg00810710	cg26786615
cg07017331	cg02837935	cg14774440	cg00638020	cg06051619	cg15909443	cg21005683
cg27623451	cg01739354	cg02254709	cg17928268	cg16260421	cg20913114	cg27288829
cg17571559	cg02196592	cg14645856	cg02224138	cg26013992	cg04132418	cg02599361
cg09277709	cg16547341	cg19345662	cg03621001	cg03318695	cg00009523	cg15800276
cg27629454	cg14189678	cg19737225	cg01556552	cg04589021	cg10771931	cg13835168
